# MMP-3-mediated cleavage of OPN is involved in copper oxide nanoparticle-induced activation of fibroblasts

**DOI:** 10.1186/s12989-023-00532-y

**Published:** 2023-05-22

**Authors:** Yuanbao Zhang, Yiqun Mo, Yue Zhang, Jiali Yuan, Qunwei Zhang

**Affiliations:** 1 Department of Epidemiology and Population Health, School of Public Health and Information Sciences, University of Louisville, 485 E. Gray Street, Louisville, KY 40202 USA; 2 Northwestern University Feinberg School of Medicine, Chicago, IL 60611 USA

**Keywords:** Copper oxide nanoparticles, MMP-3, Osteopontin, Triple co-culture system, Fibroblast activation

## Abstract

**Background:**

Copper oxide nanoparticles (Nano-CuO) are one of the most produced and used nanomaterials. Previous studies have shown that exposure to Nano-CuO caused acute lung injury, inflammation, and fibrosis. However, the mechanisms underlying Nano-CuO-induced lung fibrosis are still unclear. Here, we hypothesized that exposure of human lung epithelial cells and macrophages to Nano-CuO would upregulate MMP-3, which cleaved osteopontin (OPN), resulting in fibroblast activation and lung fibrosis.

**Methods:**

A triple co-culture model was established to explore the mechanisms underlying Nano-CuO-induced fibroblast activation. Cytotoxicity of Nano-CuO on BEAS-2B, U937* macrophages, and MRC-5 fibroblasts were determined by alamarBlue and MTS assays. The expression or activity of MMP-3, OPN, and fibrosis-associated proteins was determined by Western blot or zymography assay. Migration of MRC-5 fibroblasts was evaluated by wound healing assay. MMP-3 siRNA and an RGD-containing peptide, GRGDSP, were used to explore the role of MMP-3 and cleaved OPN in fibroblast activation.

**Results:**

Exposure to non-cytotoxic doses of Nano-CuO (0.5 and 1 µg/mL) caused increased expression and activity of MMP-3 in the conditioned media of BEAS-2B and U937* cells, but not MRC-5 fibroblasts. Nano-CuO exposure also caused increased production of cleaved OPN fragments, which was abolished by MMP-3 siRNA transfection. Conditioned media from Nano-CuO-exposed BEAS-2B, U937*, or the co-culture of BEAS-2B and U937* caused activation of unexposed MRC-5 fibroblasts. However, direct exposure of MRC-5 fibroblasts to Nano-CuO did not induce their activation. In a triple co-culture system, exposure of BEAS-2B and U937* cells to Nano-CuO caused activation of unexposed MRC-5 fibroblasts, while transfection of MMP-3 siRNA in BEAS-2B and U937* cells significantly inhibited the activation and migration of MRC-5 fibroblasts. In addition, pretreatment with GRGDSP peptide inhibited Nano-CuO-induced activation and migration of MRC-5 fibroblasts in the triple co-culture system.

**Conclusions:**

Our results demonstrated that Nano-CuO exposure caused increased production of MMP-3 from lung epithelial BEAS-2B cells and U937* macrophages, which cleaved OPN, resulting in the activation of lung fibroblasts MRC-5. These results suggest that MMP-3-cleaved OPN may play a key role in Nano-CuO-induced activation of lung fibroblasts. More investigations are needed to confirm whether these effects are due to the nanoparticles themselves and/or Cu ions.

**Supplementary Information:**

The online version contains supplementary material available at 10.1186/s12989-023-00532-y.

## Background

The potential of nanomaterials to enhance performance in many technology and industry sectors has led to their rapid development, manufacture, and use in many applications [1–3]. Copper oxide nanoparticles (Nano-CuO) are one of the most produced metal oxide nanomaterials, which are widely used in a variety of applications, such as catalysts, wood protection, electronics, coating, antibacterial products, etc. due to their high surface activity, thermoelectric properties, and other special physicochemical characteristics [4–6]. While Nano-CuO provides property advantages in such products, the accompanying occupational, consumer, and environmental exposure increases the concerns of potential health issues [7].

Nano-CuO has been shown to cause oxidative stress, inflammatory response, oxidative DNA lesions, immunotoxicity, and even cell death by in vitro studies [7–10]. Several in vivo studies also showed that administration of Nano-CuO into mice induced lung epithelial cell injury, pulmonary inflammation, and finally lung fibrosis [11, 12]. Our previous study showed that Nano-CuO exposure caused upregulation of matrix metalloproteinase-3 (MMP-3) and the occurrence of epithelial–mesenchymal transition (EMT) in human lung epithelial cells, which is a cellular process playing crucial roles in disease development such as cancer and fibrosis [13]. MMP-3 is a member of the matrix metalloproteinase family, playing important roles in morphogenesis and tissue remodeling, as well as diseases such as cancer, fibrosis, etc. [14]. However, the mechanism underlying Nano-CuO-induced lung fibrosis is still unclear.

Activation of fibroblasts is commonly considered the cellular drive that leads to the development of fibrosis [15]. Activated fibroblasts have a high capacity to produce extracellular matrix proteins, which contribute to the formation of fibrotic foci and the contraction of fibrotic tissues [15, 16]. Nanoparticle-induced activation of fibroblasts has been reported in previous studies [17–25]. For example, exposure to multi-walled carbon nanotubes (MWCNTs) caused secretion of TGF-β1 from alveolar macrophages, which subsequently induced TGF-β1-dependent fibrotic response in both co-culture model (RAW264.7 and NIH 3T3 cells) and male spontaneously hypertensive rats [25]. MWCNTs exposure also induced NLRP3 inflammasome activation in human airway epithelial cells, which further mediated TGF-β independent pro-fibrotic responses in lung fibroblasts [20]. However, whether Nano-CuO exposure could cause activation of fibroblasts and the underlying mechanisms are still not fully elucidated.

Osteopontin (OPN), also named secreted phosphoprotein 1 (SPP1), is an important cytokine in body fluids and the extracellular matrix [26]. Human OPN has two well-known integrin-binding motifs, a typical RGD (Arg–Gly–Asp) motif and an SVVYGLR (Ser-Val-Val-Tyr-Gly-Leu-Arg) integrin-binding site, through which it binds integrin family such as αvβ3, playing key roles in many physiological and pathological progresses, including inflammation, cancer, COPD, asthma, and fibrosis [27–29]. Elevated expression of OPN has been observed in the lungs of patients with diseases such as asthma and idiopathic pulmonary fibrosis (IPF), and the increased OPN level is associated with the severity of these lung diseases [30–32]. Another study showed that OPN was highly expressed in bleomycin-induced lung fibrosis, while RMV-7, an αv integrin monoclonal antibody, significantly suppressed the fibrotic responses caused by bleomycin in both in vitro and in vivo models [33]. Nanoparticle-induced overexpression of OPN has also been reported. For example, OPN was highly and persistently expressed in mouse lungs responding to carbon nanotube (CNT) exposure, and knocking out of OPN had a protective effect against CNTs-induced fibrotic focus formation and fibroblast accumulation in mouse lungs [16, 34]. In addition, the bioactivity of OPN is modified by proteolytic cleavage through which OPN exposes its integrin-binding motifs that are masked in intact OPN, and cleaved OPN exerts enhanced biological activity than the intact protein [35–37]. It is reported that OPN is a substrate of MMP-3 and cleavage of OPN by MMP-3 potentiated its bioactivity [38]. Interestingly, MMP-3, a member of MMPs and an important mediator of pulmonary fibrosis, is always co-expressed with OPN in fibrotic responses [38–40]. Our previous study has shown that exposure to Nano-CuO caused increased expression and activity of MMP-3 in human lung epithelial cells [13]. This raises the intriguing possibility that MMP-3-cleaved OPN induced by Nano-CuO exposure may be involved in Nano-CuO-induced pulmonary fibrosis. Investigating the relationship between MMP-3-cleaved OPN and fibroblast activation after metal nanoparticle exposure has not been reported, which will contribute to our full understanding of the mechanisms underlying metal nanoparticle-induced pulmonary fibrosis.

In the present study, a triple co-culture model consisting of human lung epithelial cells, macrophages, and fibroblasts was established to explore the roles of MMP-3 and OPN in the fibrotic responses in vitro after Nano-CuO exposure. We hypothesized that exposure of human lung epithelial cells and macrophages to Nano-CuO would increase the expression and activity of MMP-3, which cleaved OPN to produce bioactive OPN fragments, contributing to the activation of lung fibroblasts.

## Methods

### Copper oxide nanoparticles and their characterization

Copper (II) oxide nano-powder (Nano-CuO) was gained from Sigma-Aldrich (St. Louis, MO, USA). The characteristics of Nano-CuO was described in our and other previous studies [10, 41]. Briefly, the mean diameter of Nano-CuO in the powder is 42 ± 10 nm determined by transmission electron microscopy (TEM). The specific surface area of Nano-CuO is 23 m^2^/g. Nano-CuO was suspended in physiological saline at a concentration of 100 µg/mL. To reduce agglomeration, nanoparticle suspension was ultrasonicated by an ultrasonic cleaner FS30 (Fisher Scientific, Pittsburg, PA, USA) for 10 min prior to each experiment.

### Chemicals and reagents

CellTiter 96® AQ_ueous_ Non-Radioactive Cell Proliferation Assay (MTS assay) was purchased from Promega (Madison, WI, USA), and alamarBlue™ Cell Viability Reagent (alamarBlue assay) was from Invitrogen (Eugene, OR, USA). Phorbol 12-myristate 13-acetate (PMA) was obtained from Promega (Madison, WI, USA). MMP-3 substrate β-casein was from SIGMA (Saint Louis, MO, USA).

Antibodies against β-actin (cat.# 58169, 1:2000), collagen type 1 (Col1A1, cat.# 84336, 1:1000), fibronectin (cat.# 26836, 1:1000), TGF-β (cat.# 3711, 1:1000), and horseradish peroxidase (HRP)-conjugated horse anti-mouse IgG (cat.# 7076, 1:2000) and goat anti-rabbit IgG (cat.# 7074, 1:2000) were purchased from Cell Signaling Technology (Beverly, MA, USA). Anti-MMP-3 antibody (cat.# 53015 and 52915, 1:1000) was from abcam (Cambridge, MA, USA), and anti-α-SMA (cat.# A5228, 1:1000) antibody was from SIGMA (Saint Louis, MO, USA). Anti-osteopontin (OPN) polyclonal antibody (cat.# PA5-34579, 1:1000) was produced by Invitrogen (Rockford, IL, USA) and obtained from Thermo Fisher Scientific (Waltham, MA, USA), which was used to detect both full-length OPN (66 kDa) and MMP-3-cleaved OPN (40 kDa N-terminal fragment). The immunogen to produce this antibody is an 18 amino acid peptide near the amino terminus of human OPN. All other chemicals were purchased from Fisher Scientific (Fair Lawn, NJ, USA) except when otherwise stated. All chemicals used were of analytical grade.

### Cell culture

Human U937 monocytes (cat.# CRL-1593.2), normal human bronchial epithelial cells BEAS-2B (cat.# CRL-9609), and human fibroblasts MRC-5 (cat.# CCL-171) were purchased from American Type Culture Collection (ATCC, Manassas, VA, USA). U937 and BEAS-2B cells were maintained in RPMI 1640 medium with L-glutamine, while MRC-5 cells were in Eagle's Minimum Essential Medium (EMEM), supplemented with 10% fetal bovine serum (FBS), 100 U/mL penicillin, and 100 µg/mL streptomycin (Corning, Manassas, VA, USA) in a humidified atmosphere at 37 °C and 5% CO_2_. Prior to use, U937 monocytes were differentiated into macrophages (U937*) with 100 nM PMA at 37 °C for 48 h.

### Cytotoxicity assays

The cytotoxicity of Nano-CuO in BEAS-2B, U937*, and MRC-5 cells were determined by both alamarBlue assay and MTS assay as described in our previous studies [42]. Briefly, cells were seeded in 96-well plates. After 12 h incubation, cells were treated with 0.5, 1, 2, 5, and 10 μg/mL of Nano-CuO in a total volume of 200 µL per well. Cells without treatment were used as control. After 24 h (BEAS-2B and U937* cells) or 48 h (MRC-5 cells) treatment, the cytotoxicity was determined by recording the colorimetric absorbance at 490 nm for MTS assay and fluorescence at ex530/em590 for alamarBlue assay. The cell viability was presented as the percentage of the control.

### Collection of conditioned media

4 × 10^5^ BEAS-2B or U937* cells per well were seeded in 6-well plates in 2 mL complete EMEM. After overnight culture, the cells were exposed to Nano-CuO for 12 h. The cell culture media were collected and centrifugated. For co-culture of BEAS-2B and U937*, BEAS-2B cells were seeded in 6-well plates and incubated overnight. Then U937* macrophages were added at the ratio of 1:1 or 9:1 (BEAS-2B:U937*, total 4 × 10^5^ cells per well). BEAS-2B-U937* co-culture was cultured in 2 mL complete EMEM for 12 h. Then the co-culture was exposed to 0.5 or 1 µg/mL of Nano-CuO for another 12 h. The cell culture media were collected and centrifugated, and the supernatant was used as conditioned media to culture MRC-5 fibroblasts for 0, 12, 24, 48, and 72 h. Supernatant from unexposed cells was used as a negative control.

### Triple co-culture model

The triple co-culture model was composed of three kinds of human cells: human bronchial epithelial cells (BEAS-2B), macrophages (differentiated U937), and lung fibroblasts (MRC-5), and was set according to the previous studies with little modification [21, 43–45]. Briefly, BEAS-2B cells were seeded at first in the insert of Corning Transwell®-Clear unit (24 mm diameter insert, 0.4 μm pore size, polyester membrane, Corning, NY, USA) in 2 mL complete EMEM. 1.5 mL complete medium was added to the lower chamber. After overnight incubation, the medium was aspirated, and 2 mL U937* macrophage suspension was added on top of the BEAS-2B epithelial monolayer at the ratio of 1:1 or 9:1 (BEAS-2B:U937*). The total BEAS-2B and U937* cells were 2 × 10^5^ cells per insert. U937* macrophages were allowed to attach for 12 h, then the medium was changed. The cells were incubated for another 24 h.

MRC-5 cells were seeded in 6-well plates at a density of 1 × 10^5^ cells/mL in a volume of 2 mL/well and incubated for 48 h. Then, the inserts above were placed in the wells containing MRC-5 cells, and 1 mL of complete EMEM was added to the insert and 1.5 mL in the lower chamber. After the triple co-culture model was incubated for 12 h, BEAS-2B and U937* cells in the inserts were exposed to 1 µg/mL of Nano-CuO for 12 h. After exposure, the inserts were taken out and MRC-5 cells in the lower chamber were incubated for another 36 h. For studying the role of MMP-3-cleaved OPN in the activation and migration of MRC-5 fibroblasts in the triple co-culture system after Nano-CuO exposure, MRC-5 fibroblasts were pretreated with GRGDSP (Gly-Arg-Gly-Asp-Ser-Pro) peptide (200 µg/mL, SIGMA, St. Louis, MO, USA) for 2 h before BEAS-2B and U937* cells in the inserts were exposed to 1 µg/mL of Nano-CuO for another 12 h. GRGDSP is a soluble integrin-blocking RGD-based peptide, which can interrupt the binding of cleaved OPN to cell surface integrins.

### Protein extraction and Western blot

Total proteins were extracted using RIPA lysis buffer (Santa Cruz, CA, USA). Protein concentration was determined by Bradford protein assay (Bio-Rad, Hercules, CA, USA). Western blot was performed as described previously [13]. Briefly, proteins were separated on SDS-PAGE and transferred on Immun-Blot™ polyvinylidene fluoride (PVDF) membrane (Bio-Rad, Hercules, CA, USA). After blocking with 5% fat-free milk in 1 × TBS with 0.05% tween-20 for 2 h, membranes were incubated with the primary antibody in 5% BSA at 4 °C overnight with gentle shaking. After washing, membranes were incubated with HRP-conjugated secondary antibody for 1–2 h at room temperature. The bands were detected by using SuperSignal™ West Pico PLUS Chemiluminescent Substrate (Thermo Scientific, Rockford, IL, USA) followed by exposure to CL-XPosure™ film (Thermo Scientific). Films were scanned by using an HP Officejet Pro8500 printer and quantified by using NIH ImageJ software (https://imagej.nih.gov/ij/).

To determine the protein levels of MMP-3 and cleaved OPN, the media were collected and concentrated by Amicon® Ultra centrifugal filter units (with Ultracel-10 K membrane) ten times before electrophoresis. The gel stained with 0.1% Coomassie Brilliant Blue R-250 (Bio-Rad) was used as a loading reference.

### β-casein zymography assay

MMP-3 activity was measured by β-casein zymography assay as described previously with modifications [13]. Briefly, cells were seeded in 6-well plates in serum-free RPMI1640. After Nano-CuO treatment, the media were collected and concentrated by Amicon® Ultra centrifugal filter devices (with Ultracel-10 K membrane, Millipore, Billerica, MA, USA) ten times. After electrophoresis on 10% SDS–polyacrylamide gel with 1 mg/mL β-casein under non-reducing condition, gels were washed twice (30 min each) in 2.5% Triton X-100 solution, and incubated in calcium assay buffer (pH 7.5) containing NaCl (150 mM), CaCl_2_ (10 mM), ZnCl_2_ (5 μM), and Triton X-100 (1%) at 37 °C for 36 h. After staining with 0.1% Coomassie Brilliant Blue R-250 (Bio-Rad), the gels were destained in 10% acetic acid until clear bands were observed against the background of Coomassie Blue-stained gel.

### Transfection of BEAS-2B and U937* cells with MMP-3 siRNA

Transfection was performed as described in our previous study with modifications [13]. Briefly, BEAS-2B cells were seeded in the inserts and incubated for 12 h, then U937* cells were seeded at a ratio of 1:1 or 9:1 (BEAS-2B:U937*) onto the BEAS-2B cell layer in antibiotic-free medium supplemented with 10% FBS and allowed to attach. Cells were then transfected with a mixture of 6 µL of TurboFectin 8.0 Transfection Reagent (Origene, Rockville, MD, USA) and 30 nM of MMP-3 siRNA (Ambion, Carlsbad, CA, USA) in a total volume of 1 mL antibiotic-free and FBS-free medium for 6 h. Afterward, 1 mL medium containing 2 times FBS and antibiotics was added, and the cells were incubated for another 12 h. Silencer™ Select Negative Control No. 2 siRNA (Ambion, Carlsbad, CA, USA) was used as a negative control. After exposure to Nano-CuO for 12 h, the conditioned media were collected and the roles of MMP-3 in the cleavage of OPN and activation of fibroblasts were determined.

### Wound healing assay

Fibroblast migration ability was evaluated by in vitro wound healing assay. Briefly, MRC-5 fibroblasts were seeded in 12-well plates and incubated in EMEM medium until 70–80% confluency. The wound was carefully made by scratching a straight line in the monolayer using a pipette tip. A second scratch was made perpendicular to the first one and the wells were washed twice with 1 × PBS to remove any detached cells. After treatment with conditioned media for 48 h, the cell image was captured by a microscope (Nikon, Japan) and the wound closure area was calculated by using NIH ImageJ software (https://imagej.nih.gov/ij/), which was used to determine the migration ability of fibroblasts.

### Statistical analysis

Results were expressed as the mean ± standard error (SE). The differences among groups with one independent variable were evaluated by one-way analysis of variance (ANOVA) with the Bonferroni post-hoc test. The differences among groups with two independent variables were evaluated by two-way ANOVA with the Holm-Sidak test. All analyses were carried out using SigmaPlot 13.0 software (Systat Software, San Jose, CA, USA). A *p *value < 0.05 was deemed statistically significant.

## Results

### Cytotoxic effects of Nano-CuO on BEAS-2B, U937*, and MRC-5 cells

**Fig. 1:**
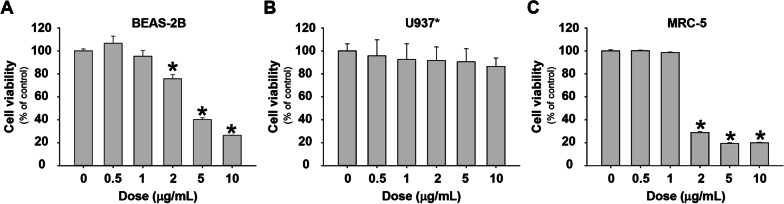
Cytotoxicity of Nano-CuO on BEAS-2B cells, U937-derived macrophages (U937*), and MRC-5 fibroblasts. BEAS-2B (**a**), U937* macrophages (**b**), and MRC-5 fibroblasts (**c**) were seeded into 96-well plates and treated with 0.5, 1, 2, 5, and 10 µg/mL of Nano-CuO for 24 h (**a** and **b**) and 48 h (**c**), respectively. Cells without treatment were used as control. The cytotoxicity was determined by alamarBlue assay. Data represent mean ± SE (n = 6). * Significant difference as compared to the control group, *p* < 0.05

The cytotoxicity of Nano-CuO on BEAS-2B, U937*, and MRC-5 cells was at first determined by alamarBlue assay after the cells were exposed to 0, 0.5, 1, 2, 5, and 10 µg/mL of Nano-CuO for 24 h (BEAS-2B and U937*) or 48 h (MRC-5). The results showed that exposure of BEAS-2B to 2 µg/mL or higher concentrations of Nano-CuO for 24 h caused a significant decrease in cell viability (Fig. 1a). However, no significant cytotoxicity was observed in U937* cells after 24 h Nano-CuO exposure at all experimental doses (Fig. 1b). Exposure of MRC-5 cells to 2 µg/mL or higher concentrations of Nano-CuO for 48 h also caused significant cytotoxicity (Fig. 1c). These results were further confirmed by MTS assay (data not shown). Non-cytotoxic doses (≤ 1 µg/mL) were chosen for the following in vitro experiments.

### Exposure to Nano-CuO increased the expression and activity of MMP-3 in the conditioned media of BEAS-2B and U937* cells, but not MRC-5 fibroblasts

**Fig. 2:**
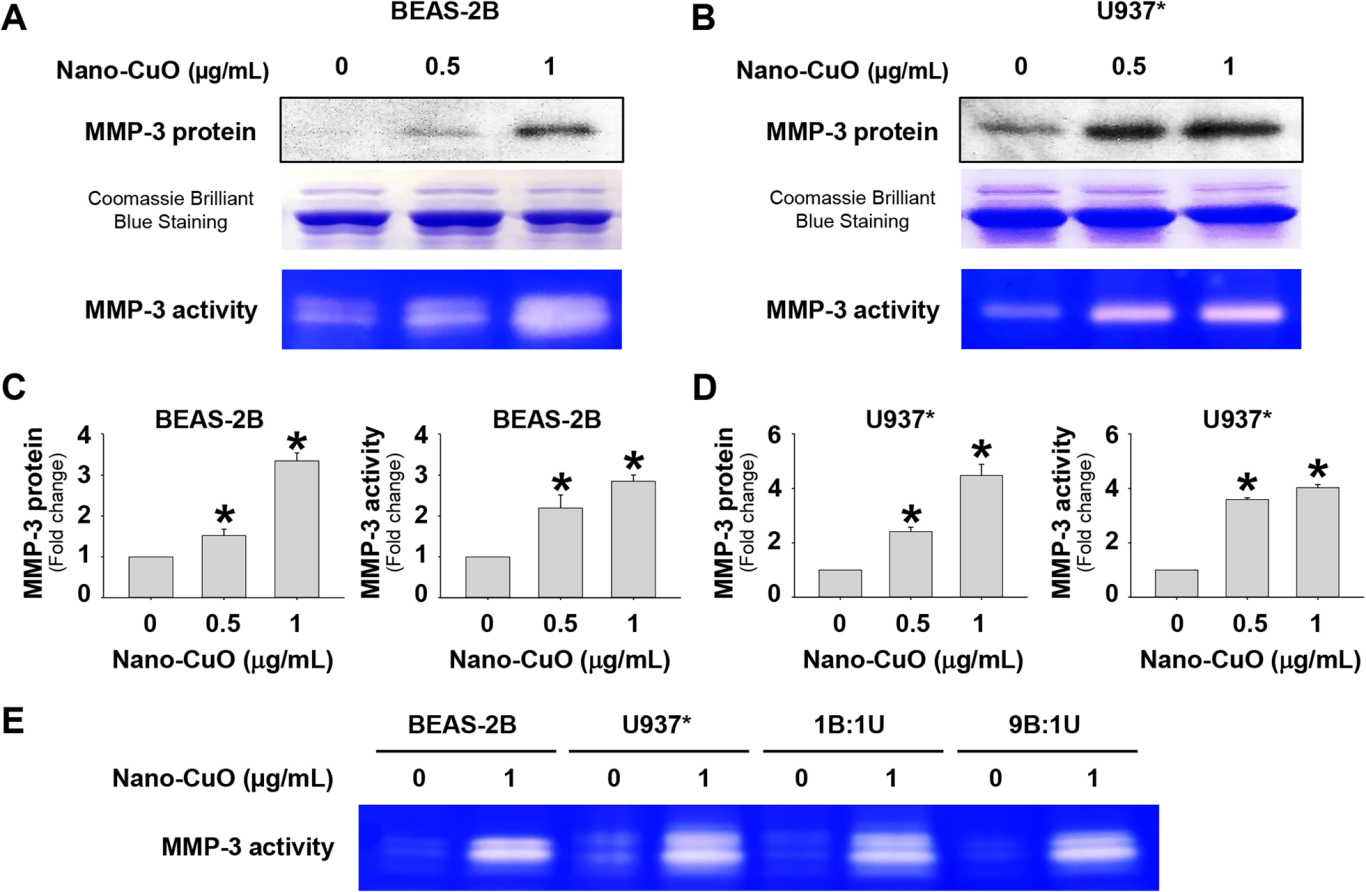
Increased expression and activity of MMP-3 in the conditioned media of BEAS-2B cells and U937* macrophages exposed to Nano-CuO. BEAS-2B cells (**a** and **c**) and U937* macrophages (**b** and **d**) were exposed to 0.5 and 1 µg/mL of Nano-CuO for 12 h. After treatment, the conditioned media were collected. Cells without treatment were used as control. MMP-3 protein level in the conditioned media was detected by Western blot, while its activity was detected by β-casein zymography. Equal protein loading was verified by Coomassie Brilliant Blue staining. The activity of MMP-3 was also determined in the conditioned media from co-culture system (**e**). **a**, **b**, and **e** were the results of a single experiment, while **c** and **d** were normalized band densitometry readings averaged from three independent experiments. Data represent mean ± SE (n = 3). * Significant difference as compared to the control group, *p* < 0.05

The effects of Nano-CuO on MMP-3 expression and activity in the conditioned media of BEAS-2B, U937*, and MRC-5 cells were detected by Western blot and β-casein zymography assay, respectively. Our previous time-dependent study has shown that exposure to Nano-CuO caused an increase in the expression and activity of MMP-3 in the conditioned medium of BEAS-2B cells, and the peak occurred at 12 h after Nano-CuO exposure [13]. Therefore, in the present study, BEAS-2B, U937*, and MRC-5 cells were exposed to 0, 0.5, and 1 µg/mL of Nano-CuO for 12 h, and the expression and activity of MMP-3 in the media were measured after exposure. The results demonstrated that exposure of BEAS-2B cells to Nano-CuO caused a dose-dependent increase in the expression of MMP-3, with a 3-4 fold increase when the cells were exposed to 1 µg/mL of Nano-CuO for 12 h (Fig. 2a, c). Similar results were also observed in U937* cells after Nano-CuO exposure (Fig. 2b, d). The MMP-3 activity detected by β-casein zymography assay was consistent with its protein expression results (Fig. 2a–d). Exposure to 1 µg/mL of Nano-CuO caused a 3-4 fold increase in MMP-3 activity in the media of both BEAS-2B and U937* cells. However, in MRC-5 fibroblasts, Nano-CuO exposure did not cause any change in the expression of MMP-3 (Additional file 1a).

To explore the effects of Nano-CuO exposure on the activity of MMP-3 in the co-culture system, BEAS-2B and U937* cells were co-cultured at the ratio of 1:1 and 9:1 (BEAS-2B:U937*) and then exposed to 1 µg/mL of Nano-CuO for 12 h. After exposure, MMP-3 activity in the media was measured by using β-casein zymography. The results demonstrated that Nano-CuO exposure induced significant increase in the MMP-3 activity in both 1:1 and 9:1 co-culture systems (Fig. 2e), which were similar to those in mono-culture models.

### Exposure to Nano-CuO caused upregulation of OPN in BEAS-2B and U937* cells, but not in MRC-5 fibroblasts

**Fig. 3:**
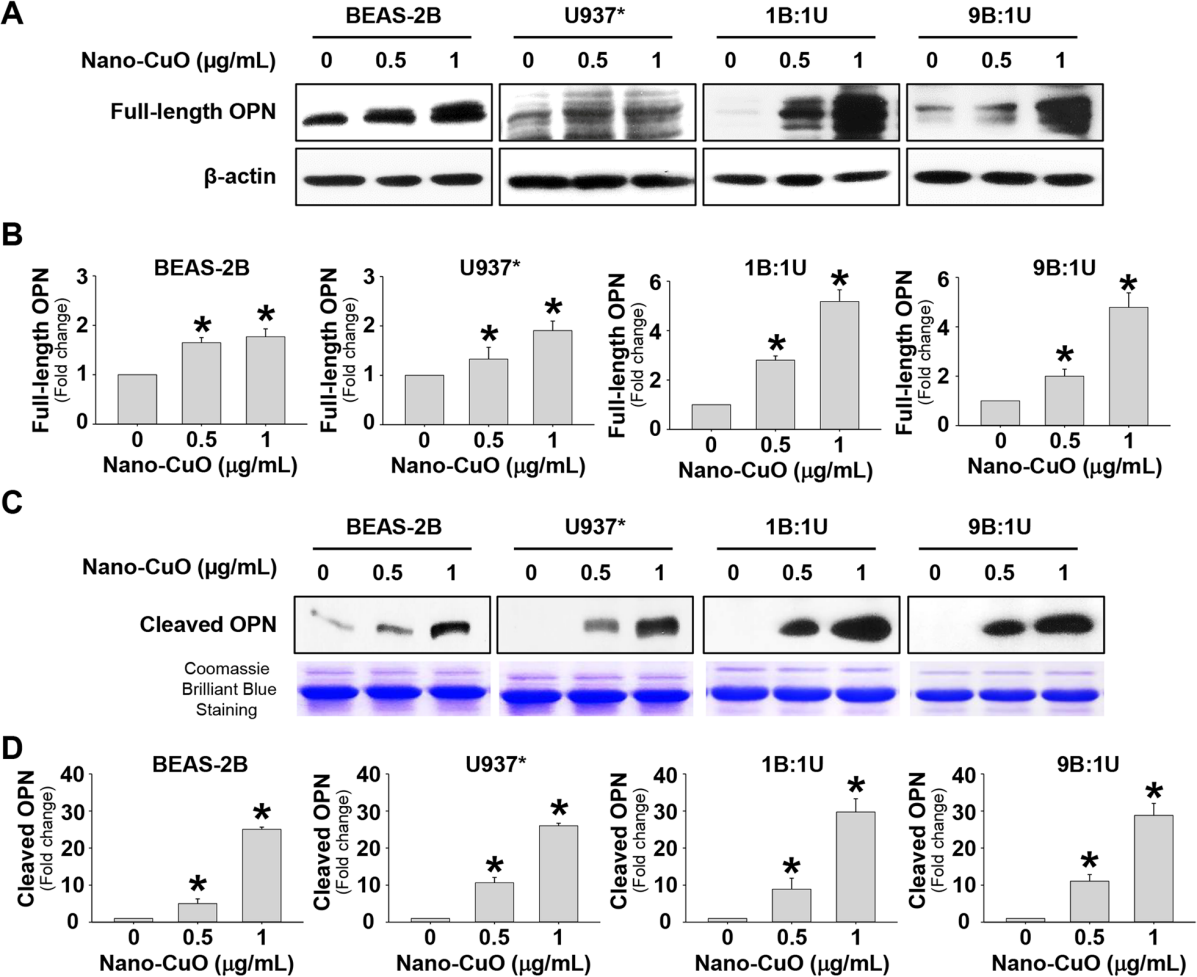
Increased expression of OPN in BEAS-2B cells and U937* macrophages exposed to Nano-CuO. BEAS-2B cells, U937* macrophages, or co-culture of BEAS-2B and U937* macrophages at a ratio of 1:1 or 9:1 (BEAS-2B:U937*) were exposed to 0.5 and 1 µg/mL of Nano-CuO for 12 h. After treatment, the cells and conditioned media were collected. Cells without treatment were used as control. Full-length OPN (66 kDa) in the cells and cleaved OPN (40 kDa N-terminal fragment) in the conditioned media were detected by Western blot. Equal protein loading was verified by β-actin expression (**a** and **b**) or Coomassie Brilliant Blue staining (**c** and **d**). **a** and **c** were the results of a single experiment. **b** and **d** were results of three independent experiments. Data represent mean ± SE (n = 3). * Significant difference as compared to the control group, *p* < 0.05

To study whether exposure to Nano-CuO could cause upregulation of OPN, BEAS-2B, U937*, and MRC-5 cells were treated with 0.5 and 1 µg/mL of Nano-CuO for 12 h, and the expression of OPN was determined by Western blot. The results showed that Nano-CuO exposure induced increased expression of full-length OPN in BEAS-2B and U937* cells (Fig. 3a, b), indicating Nano-CuO exposure caused OPN upregulation in the cells. In addition, the MMP-3-cleaved OPN fragment in the conditioned media from BEAS-2B and U937* cells were also increased (Fig. 3c, d), although the alteration of full-length OPN in the conditioned media was not observed (Additional file 2). In MRC-5 fibroblasts, Nano-CuO exposure did not induce an increased level of cleaved OPN in the conditioned media (Additional file 1b).

Nano-CuO-induced OPN production was also determined in the co-culture system by Western blot. BEAS-2B and U937* cells were seeded at a ratio of 1:1 or 9:1 (BEAS-2B:U937*) and exposed to 0.5 or 1 µg/mL of Nano-CuO for 12 h. In both 1:1 and 9:1 co-culture systems, Nano-CuO exposure caused increased expression in full-length OPN in the cells and increased production of cleaved OPN in the media (Fig. 3a-d).

### The role of MMP-3 in Nano-CuO-induced production of cleaved OPN

**Fig. 4:**
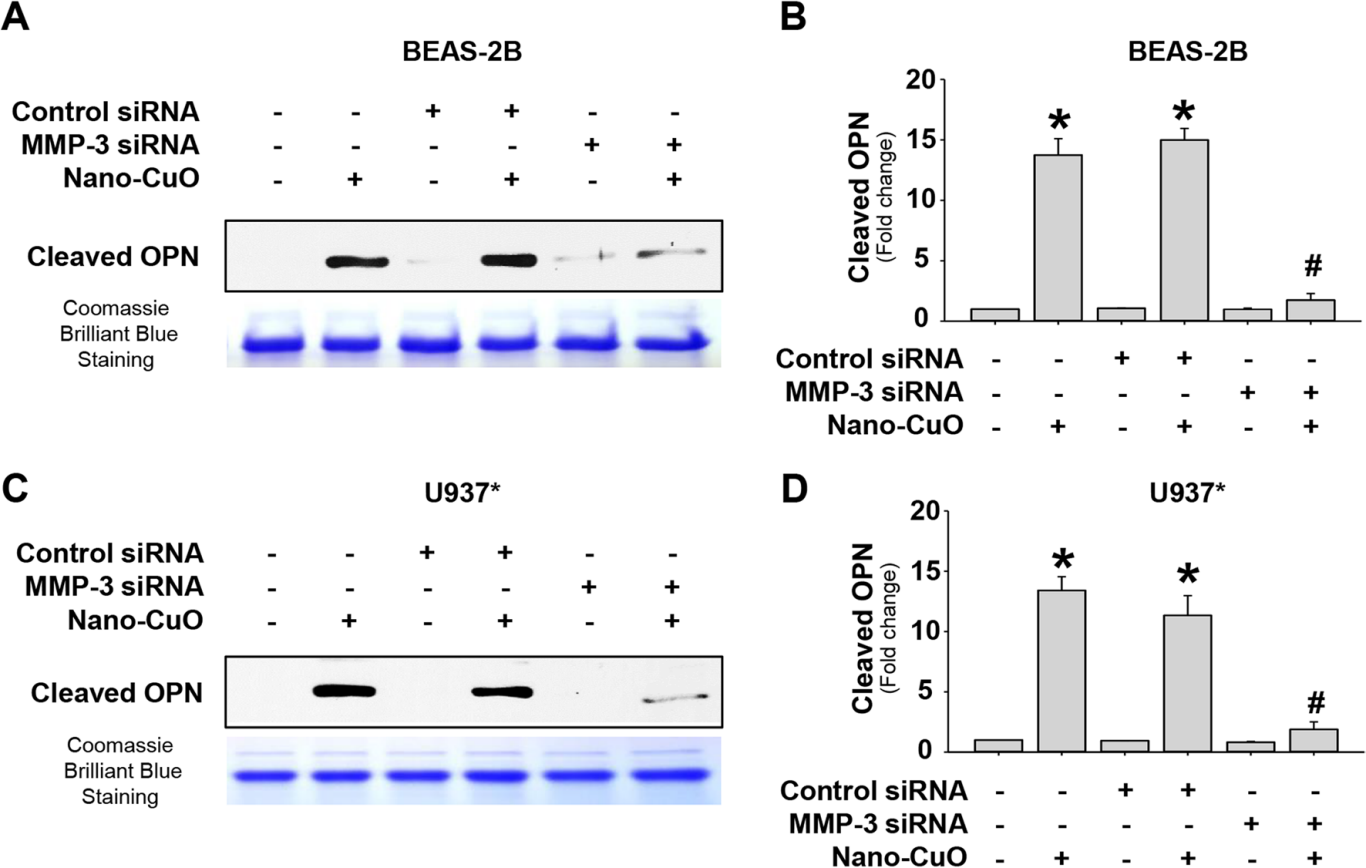
The role of MMP-3 on Nano-CuO-induced production of cleaved OPN in BEAS-2B cells and U937* macrophages. BEAS-2B cells (**a** and **b**) and U937* macrophages (**c** and **d**) were transfected with 30 nM of MMP-3 siRNA or Negative Control No. 2 siRNA. After transfection, the cells were exposed to 1 µg/mL of Nano-CuO for 12 h. The conditioned media were collected to detect the level of cleaved OPN (40 kDa N-terminal fragment) by Western blot. Equal protein loading was verified by Coomassie Brilliant Blue staining. **a** and **c** were the results of a single experiment. **b** and **d** were normalized band densitometry readings averaged from three independent experiments. Data represent mean ± SE (n = 3). * Significant difference as compared to the control group, *p* < 0.05; # Significant difference as compared to the Nano-CuO-treated group transfected with Negative Control No. 2 siRNA, *p* < 0.05

To determine the role of MMP-3 in Nano-CuO-induced increased production of cleaved OPN, BEAS-2B or U937* cells were transfected with 30 nM of MMP-3 siRNA and then exposed to 1 µg/mL of Nano-CuO for 12 h. Media were collected for the detection of MMP-3 protein and cleaved OPN by Western blot. Our results showed that MMP-3 siRNA transfection significantly reduced the level of Nano-CuO-induced MMP-3 protein in the conditioned media of BEAS-2B cells [13] and U937* macrophages (Additional file 3). MMP-3 siRNA transfection also significantly abolished the increased production of cleaved OPN induced by Nano-CuO exposure in both BEAS-2B (Fig. 4a, b) and U937* (Fig. 4c, d) cell culture media. However, the upregulation of full-length OPN in the cells exposed to Nano-CuO was not affected by MMP-3 siRNA transfection (data not shown).

### Direct Nano-CuO exposure did not activate MRC-5 fibroblasts

**Fig. 5:**
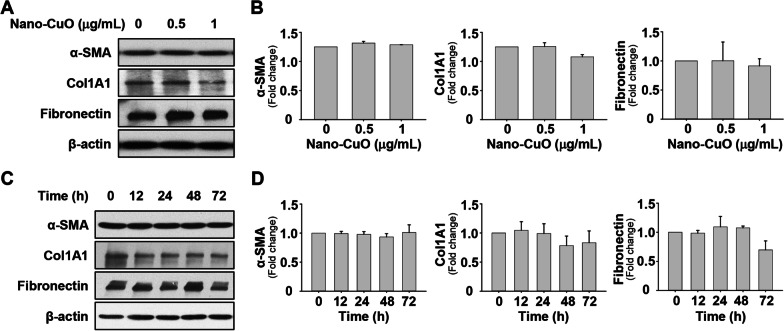
Direct Nano-CuO exposure had no effects on activation of MRC-5 fibroblasts. MRC-5 cells were treated with 0.5 and 1 µg/mL of Nano-CuO for 48 h (**a** and **b**) or with 1 µg/mL of Nano-CuO for 12, 24, 48, and 72 h (**c** and **d**). Cells without treatment were used as control. Proteins were isolated from the cells to detect the expression of α-SMA, Col1A1, and fibronectin. **a** and **c** were the results of a single Western blot experiment. **b** and **d** were the average expression levels of α-SMA, Col1A1, and fibronectin normalized to β-actin from three independent experiments. Data represent mean ± SE (n = 3)

To explore whether exposure to Nano-CuO would activate fibroblasts, MRC-5 cells were treated with 0, 0.5, and 1 µg/mL of Nano-CuO for 48 h, and the expression of α-SMA, Col1A1, and fibronectin was measured by Western blot. The results showed that direct exposure to Nano-CuO for 48 h did not cause significant alterations in the expression of α-SMA, Col1A1, and fibronectin (Fig. 5a, b). For the time-response study, MRC-5 cells were exposed to 1 µg/mL of Nano-CuO for 0, 12, 24, 48, and 72 h. The results demonstrated that Nano-CuO also did not activate MRC-5 cells at all the time points after Nano-CuO treatment (Fig. 5c, d).

### MRC-5 fibroblasts were activated when cultured in conditioned media from Nano-CuO-exposed BEAS-2B, U937* or co-culture of BEAS-2B and U937* cells

**Fig. 6:**
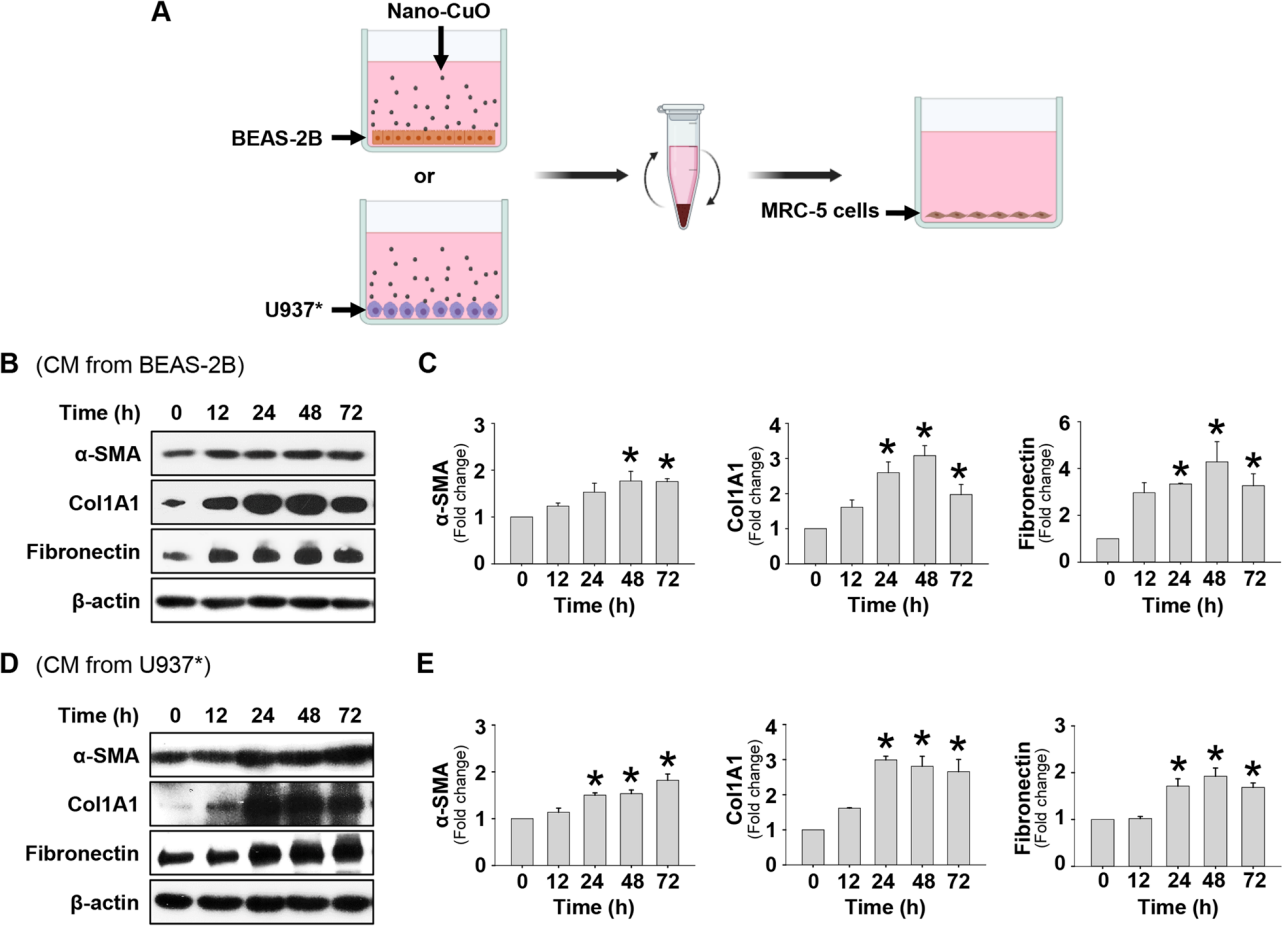
Activation of MRC-5 fibroblasts cultured in the conditioned media from Nano-CuO-exposed BEAS-2B or U937* cells. **a** is the experimental protocol. BEAS-2B or U937* cells were treated with 1 µg/mL of Nano-CuO for 12 h and the conditioned media (CM) were collected to culture MRC-5 fibroblasts. After 0, 12, 24, 48, and 72 h culture, the MRC-5 fibroblasts were collected for protein isolation and Western blot. **b** and **d** were the results of a single Western blot experiment. **c** and **e** were the average expression levels of α-SMA, Col1A1, and fibronectin normalized to β-actin from three independent experiments. Data represent mean ± SE (n = 3). * Significant difference as compared to the control group, *p* < 0.05. Graphics in **a** were created with BioRender (https://BioRender.com)

To investigate whether some mediators, such as MMP-3 and cleaved OPN, released from Nano-CuO-exposed BEAS-2B or U937*, could activate MRC-5 cells, BEAS-2B or U937* cells were treated with 1 µg/mL of Nano-CuO for 12 h and the conditioned media were collected. MRC-5 fibroblasts were then cultured in the conditioned media for 0, 12, 24, 48, and 72 h (Fig. 6a). The results demonstrated that when MRC-5 fibroblasts were cultured in the conditioned media from Nano-CuO-exposed BEAS-2B or U937* cells, MRC-5 cells were activated, which was reflected by increased expression of α-SMA, Col1A1, and fibronectin (Fig. 6b–e).

**Fig. 7:**
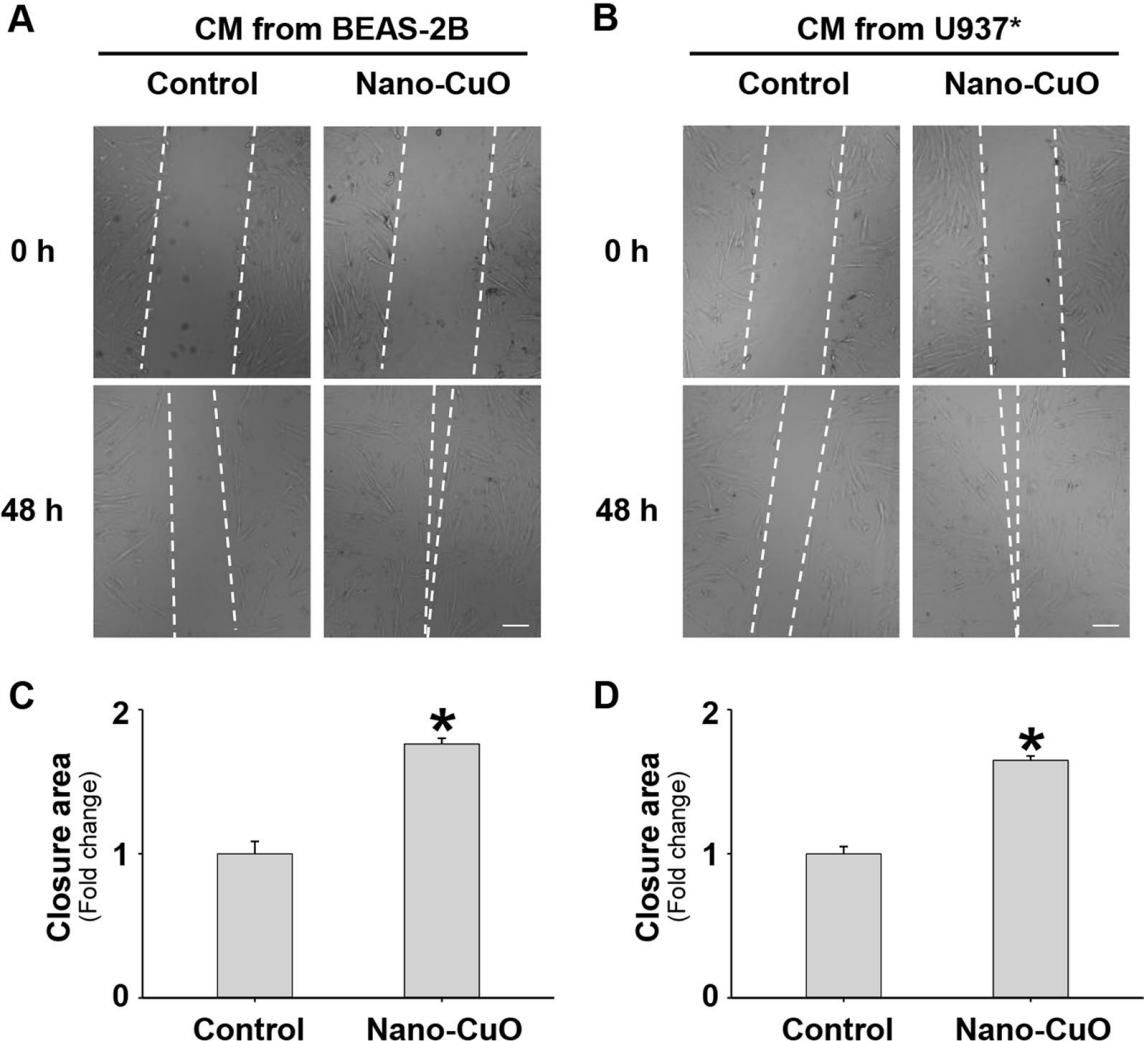
Conditioned media from Nano-CuO-exposed BEAS-2B or U937* cells enhanced migration of unexposed MRC-5 fibroblasts. BEAS-2B or U937* cells were treated with 0 or 1 µg/mL of Nano-CuO for 12 h and the conditioned media (CM) were collected to culture MRC-5 fibroblasts for 48 h. Migration of MRC-5 cells was detected by wound healing assay. **a** and **b** were representative images. **c** and **d** were the average closure area from three independent experiments. Scale bars in **a** and **b** represent 200 µm. Data represent mean ± SE (n = 3). * Significant difference as compared to the control group, *p* < 0.05

Migration of MRC-5 fibroblasts was also evaluated by wound healing assay after the cells were cultured in the conditioned media collected from Nano-CuO-exposed BEAS-2B or U937* culture. The results showed that cultured in the conditioned media from Nano-CuO-exposed BEAS-2B or U937* culture significantly promoted the migration of MRC-5 fibroblasts (Fig. 7 and Additional file 4).

**Fig. 8:**
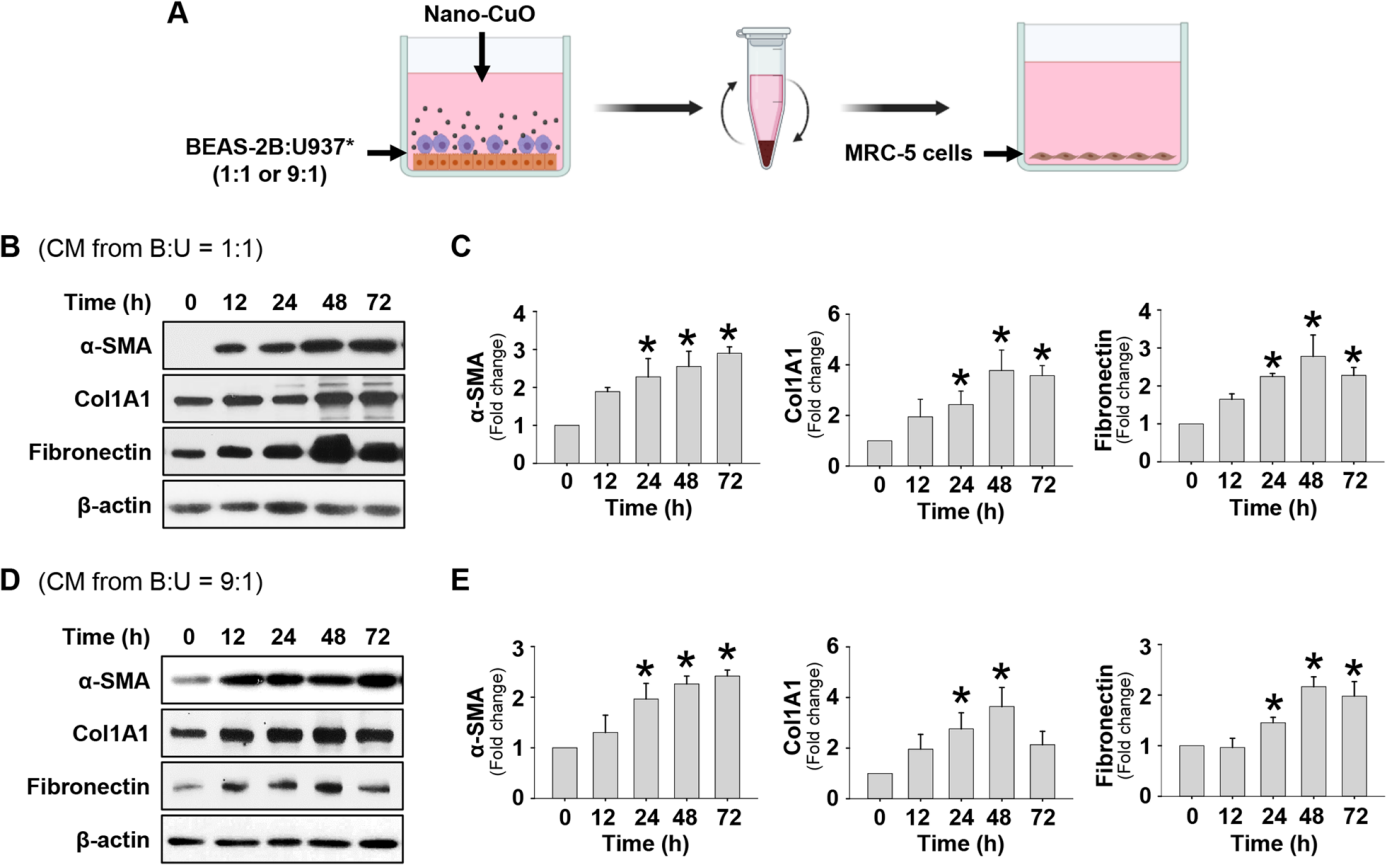
Cultured in the conditioned media from Nano-CuO-exposed co-culture of BEAS-2B and U937* cells caused activation of unexposed MRC-5 fibroblasts. **a** is the experimental protocol of the co-culture experiments. Co-culture of BEAS-2B and U937* cells with ratio of 1:1 or 9:1 (BEAS-2B:U937*) was treated with 1 µg/mL of Nano-CuO for 12 h and the conditioned media (CM) were collected. MRC-5 fibroblasts were then cultured in the conditioned media for 0, 12, 24, 48, and 72 h. Proteins from MRC-5 cells were isolated to detect the expression of α-SMA, Col1A1, and fibronectin. **b** and **d** were the results of a single Western blot experiment. **c** and **e** were the average expression levels of α-SMA, Col1A1, and fibronectin normalized to β-actin from three independent experiments. Data represent mean ± SE (n = 3). * Significant difference as compared to the control group, *p* < 0.05. Graphics in **a** were created with BioRender (https://BioRender.com)

To further investigate whether cultured in the conditioned media from Nano-CuO-exposed co-culture of BEAS-2B and U937* cells could activate unexposed MRC-5 cells, BEAS-2B and U937* cells were seeded at the ratio of 1:1 or 9:1 (BEAS-2B:U937*), and the co-culture systems were exposed to 1 µg/mL of Nano-CuO for 12 h. After exposure, the conditioned media were collected. MRC-5 fibroblasts were then cultured in the conditioned media for 0, 12, 24, 48, and 72 h (Fig. 8a). The results demonstrated that cultured in the conditioned media from co-culture of BEAS-2B and U937* (1:1 or 9:1) induced MRC-5 activation with increased expression of α-SMA, Col1A1, and fibronectin (Fig. 8b–e).

### Indirect exposure to Nano-CuO caused activation of MRC-5 fibroblasts in a triple co-culture system

**Fig. 9:**
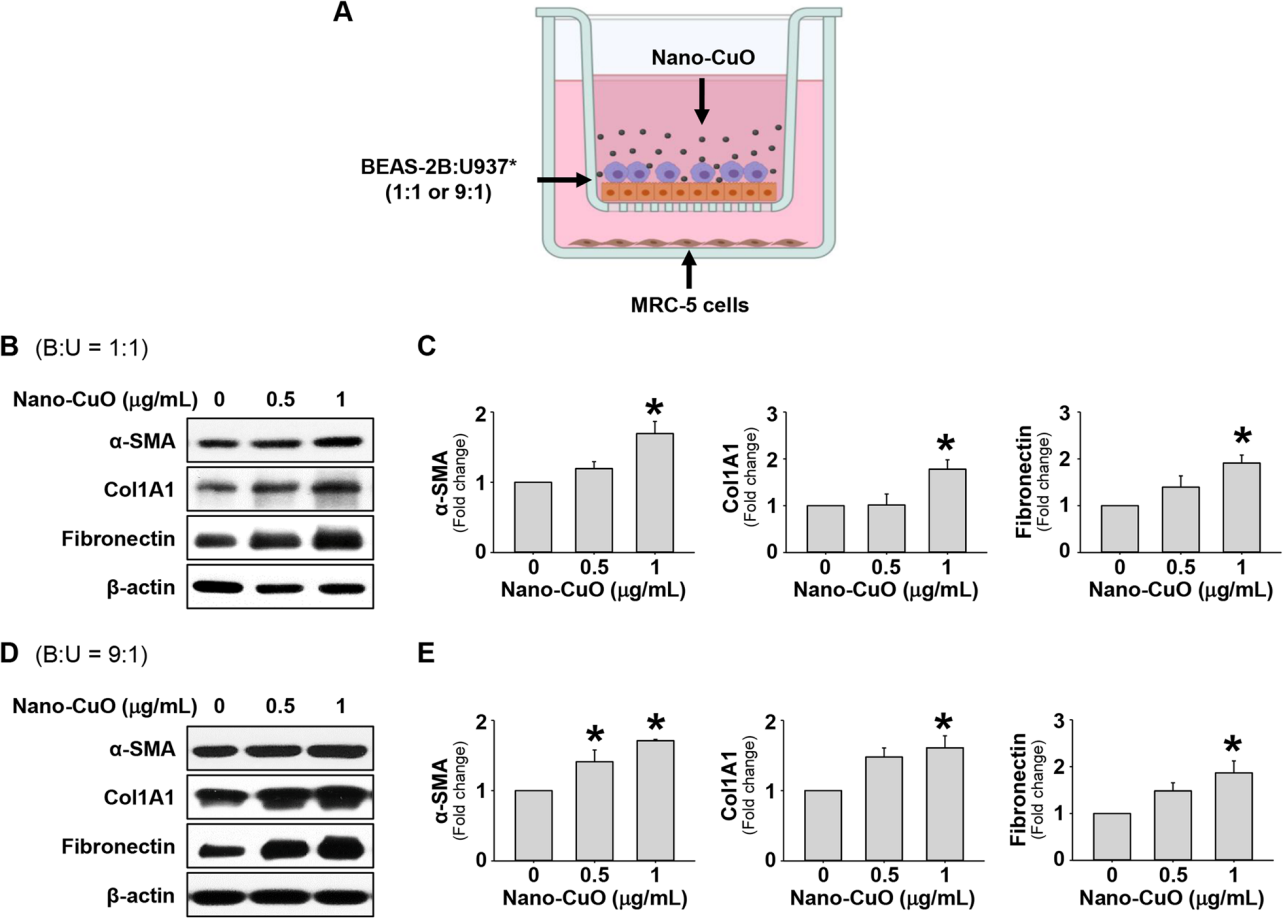
Indirect exposure to Nano-CuO caused activation of MRC-5 fibroblasts in a triple co-culture system. **a** is the experimental protocol for the triple co-culture experiments. BEAS-2B cells and U937* cells were seeded at the ratio of 1:1 (**b** and **c**) or 9:1 (**d** and **e**) (BEAS-2B:U937*) in the inserts of Transwell^®^ units and MRC-5 cells were seeded in the lower chambers of the units. After establishment of the triple co-culture system, BEAS-2B and U937* cells were exposed to 0, 0.5 and 1 µg/mL of Nano-CuO for 12 h, then the inserts were taken out. MRC-5 cells in the lower chamber were incubated for another 36 h and proteins were isolated to detect the expression of α-SMA, Col1A1, and fibronectin. **b** and **d** were the results of a single Western blot experiment. **c** and **e** were the average expression levels of α-SMA, Col1A1, and fibronectin normalized to β-actin from three independent experiments. Data represent mean ± SE (n = 3). * Significant difference as compared to the control group, *p* < 0.05. Graphics in **a** were created with BioRender (https://BioRender.com)

Next, we explored whether indirect exposure to Nano-CuO could activate MRC-5 cells in a triple co-culture system. In our triple co-culture system, BEAS-2B and U937* cells were seeded at a ratio of 1:1 or 9:1 (BEAS-2B:U937*) in the inserts of Transwell® units, and MRC-5 cells were seeded in the lower chambers of the units (Fig. 9a). After the establishment of the triple co-culture system, BEAS-2B and U937* cells were exposed to 0, 0.5 and 1 µg/mL of Nano-CuO for 12 h, then the inserts were taken out. MRC-5 cells in the lower chamber were incubated continuously for another 36 h. After incubation, proteins from the MRC-5 cells were isolated for Western blot to detect the expression levels of α-SMA, Col1A1, and fibronectin. The results showed that in both models, indirect exposure to Nano-CuO led to a significant activation of MRC-5 fibroblasts in the lower chamber of the Transwell® unit, which was reflected by the increased expression of α-SMA, Col1A1, and fibronectin (Fig. 9b–e).

### The role of MMP-3 in Nano-CuO-induced activation of MRC-5 fibroblasts in the triple co-culture system

**Fig. 10:**
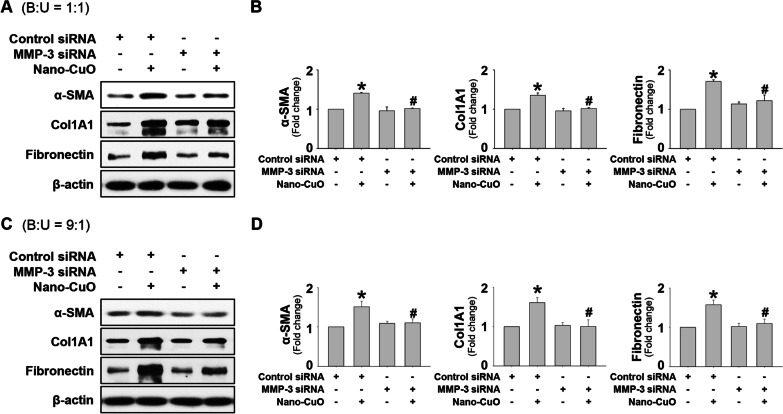
The role of MMP-3 on Nano-CuO-induced activation of MRC-5 fibroblasts in the triple co-culture system. BEAS-2B and U937* cells were seeded at the ratio of 1:1 (**a** and **b**) or 9:1 (**c** and **d**) (BEAS-2B:U937*) in the inserts of Transwell^®^ units and transfected with 30 nM of MMP-3 siRNA or Negative Control No. 2 siRNA. After transfection, the cells were exposed to 1 µg/mL of Nano-CuO for 12 h. After exposure, the inserts were taken out and the MRC-5 fibroblasts seeded in the lower chamber were incubated for another 36 h. The proteins isolated from MRC-5 fibroblasts were used to detect the expression levels of α-SMA, Col1A1, and fibronectin by Western blot. **a** and **c** were the results of a single Western blot experiment. **b** and **d** were the average expression level of α-SMA, Col1A1, and fibronectin normalized to β-actin from three independent experiments. Data represent mean ± SE (n = 3). * Significant difference as compared to the control group, *p* < 0.05; # Significant difference as compared to the Nano-CuO-treated group transfected with Negative Control No. 2 siRNA, *p* < 0.05

**Fig. 11:**
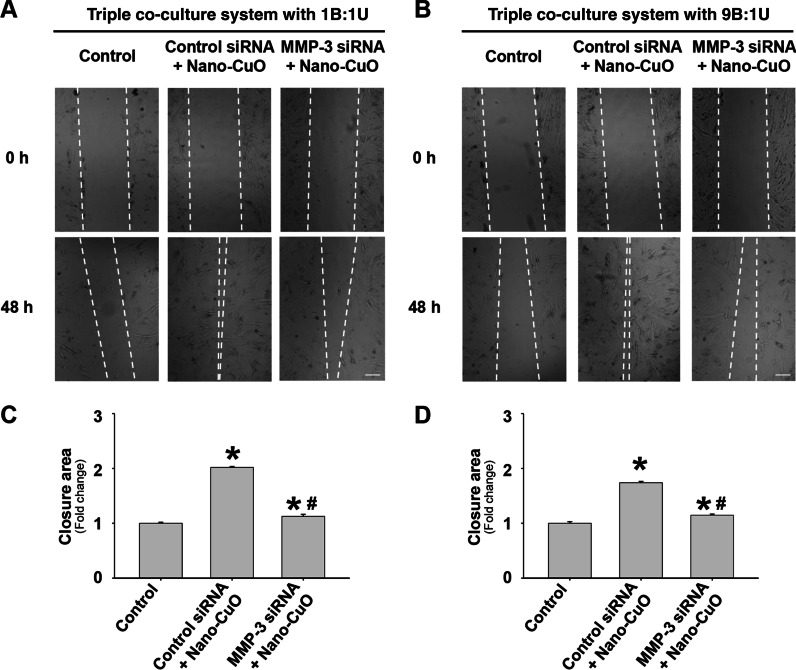
MMP-3 siRNA transfection attenuated Nano-CuO-induced migration of MRC-5 fibroblasts in the triple co-culture system. BEAS-2B and U937* cells were seeded at a ratio of 1:1 (**a** and **c**) or 9:1 (**b** and **d**) (BEAS-2B:U937*) in the inserts of Transwell^®^ units and transfected with 30 nM of MMP-3 siRNA or Negative Control No. 2 siRNA. After transfection, the cells were exposed to 1 µg/mL of Nano-CuO for 12 h. After exposure, the inserts were taken out and the MRC-5 fibroblasts seeded in the lower chamber were incubated for another 36 h. Migration of MRC-5 cells was detected by wound healing assay. Cells without any treatments were used as the control. **a** and **b** were representative images. **c** and **d** were the average closure area from three independent experiments. Scale bars in **a** and **b** represent 200 µm. Data represent mean ± SE (n = 3). * Significant difference as compared to the control group, *p* < 0.05; # Significant difference as compared to the Nano-CuO-treated group with control siRNA transfection, *p* < 0.05

To investigate the role of MMP-3 in MRC-5 fibroblast activation in our triple co-culture model, BEAS-2B and U937* cells were transfected with 30 nM of MMP-3 siRNA and then exposed to 1 µg/mL of Nano-CuO for 12 h. After exposure, the inserts were taken out and the MRC-5 fibroblasts seeded in the lower chamber were incubated continuously for another 36 h. The proteins isolated from MRC-5 fibroblasts were used to detect the expression of α-SMA, Col1A1, and fibronectin by Western blot. Negative Control No. 2 siRNA was used as a negative control. The results showed that MMP-3 siRNA transfection in BEAS-2B and U937* cells significantly suppressed the activation of MRC-5 cells, which was reflected by decreased expression of α-SMA, Col1A1, and fibronectin (Fig. 10a-d). In addition, increased migration of MRC-5 fibroblasts in the triple co-culture system was also significantly attenuated by MMP-3 siRNA transfection (Fig. 11a–d).

### The role of MMP-3-cleaved OPN in the activation of unexposed MRC-5 fibroblasts in the triple co-culture system

**Fig. 12:**
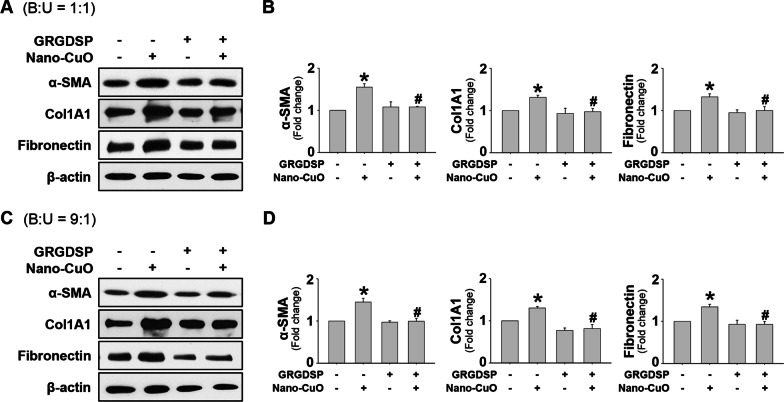
The role of MMP-3-cleaved OPN in Nano-CuO-induced activation of MRC-5 fibroblasts in the triple co-culture system. BEAS-2B and U937* cells were seeded at the ratio of 1:1 (**a** and **b**) or 9:1 (**c** and **d**) (BEAS-2B:U937*) in the inserts, and MRC-5 fibroblasts were seeded in the lower chambers of Transwell^®^ units. MRC-5 fibroblasts were pretreated with 200 µg/mL of GRGDSP peptides, then BEAS-2B and U937* cells were exposed to 1 µg/mL of Nano-CuO for 12 h. After exposure, the inserts were taken out and the MRC-5 fibroblasts were incubated for another 36 h. The proteins from MRC-5 fibroblasts were isolated to determine the activation of MRC-5 fibroblasts by Western blot. **a** and **c** were the results of a single Western blot experiment. **b** and **d** were the average expression levels of α-SMA, Col1A1, and fibronectin normalized to β-actin from three independent experiments. Data represent mean ± SE (n = 3). * Significant difference as compared to the control group, *p* < 0.05; # Significant difference as compared to the Nano-CuO-treated alone group, *p* < 0.05

**Fig. 13:**
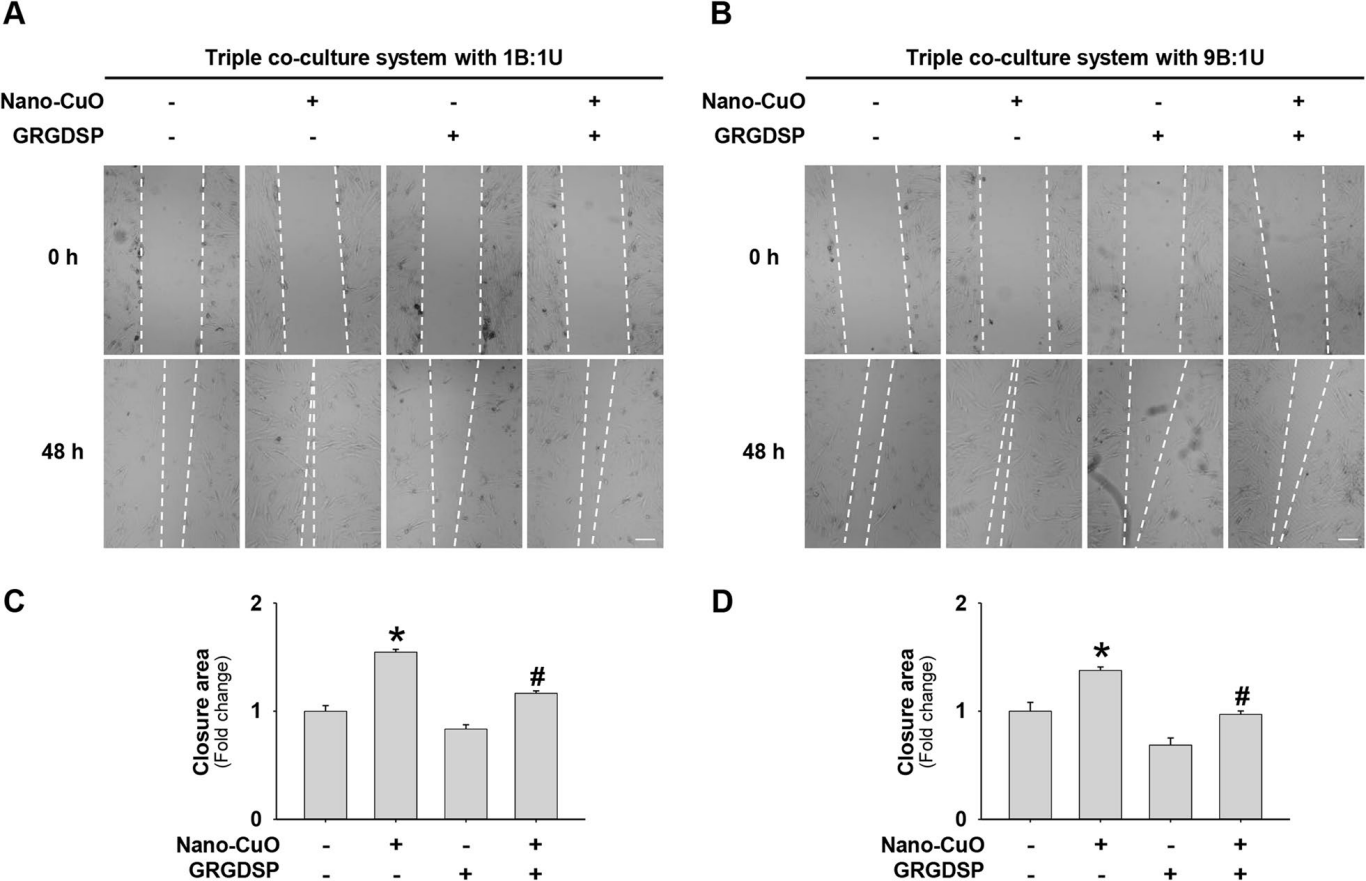
Pretreatment of GRGDSP peptides inhibited Nano-CuO-induced migration of MRC-5 fibroblasts in the triple co-culture system. BEAS-2B and U937* cells were seeded at the ratio of 1:1 (**a** and **c**) or 9:1 (**b** and **d**) (BEAS-2B:U937*) in the inserts and MRC-5 fibroblasts were seeded in the lower chambers of Transwell^®^ units. MRC-5 fibroblasts were pretreated with 200 µg/mL of GRGDSP peptides, then BEAS-2B and U937* cells were exposed to 1 µg/mL of Nano-CuO for 12 h. After exposure, the inserts were taken out and the MRC-5 fibroblasts were incubated for another 36 h. Migration of MRC-5 cells was detected by wound healing assay. **a** and **b** were representative images. **c** and **d** were the average closure area from three independent experiments. Scale bars in **a** and **b** represent 200 µm. Data represent mean ± SE (n = 3). * Significant difference as compared to the control group, *p* < 0.05; # Significant difference as compared to the Nano-CuO-treated alone group, *p* < 0.05

Our results have shown that Nano-CuO exposure induced upregulation of MMP-3 and increased production of MMP-3-cleaved OPN in BEAS-2B and U937* cells, and MMP-3 siRNA transfection significantly inhibited the production of MMP-3-cleaved OPN and activation of MRC-5 fibroblasts. To further explore whether MMP-3-cleaved OPN was involved in the activation of MRC-5 fibroblasts in the triple co-culture system after Nano-CuO exposure, MRC-5 fibroblasts were pretreated with GRGDSP peptides (200 µg/mL), which could interrupt the binding of cleaved OPN to cell surface integrins. Then BEAS-2B and U937* cells in the triple co-culture model were exposed to 1 µg/mL of Nano-CuO for 12 h. After exposure, the inserts were taken out and the MRC-5 fibroblasts seeded in the lower chamber were incubated continuously for another 36 h. The proteins isolated from MRC-5 fibroblasts were used to detect the expression of α-SMA, Col1A1, and fibronectin by Western blot. Our results revealed that GRGDSP treatment significantly attenuated the activation of MRC-5 fibroblasts in the triple co-culture system (Fig. 12a–d), suggesting that MMP-3-cleaved OPN plays a key role in the activation of MRC-5 fibroblasts. In addition, GRGDSP treatment also inhibited the migration of MRC-5 fibroblasts in the triple co-culture system (Fig. 13a–d).

## Discussion

In the present study, we established a triple co-culture model, consisting of human lung epithelial cells, macrophages, and fibroblasts, to investigate the role of MMP-3 and MMP-3-cleaved OPN in lung fibrotic response in vitro after exposure to Nano-CuO. Human lung epithelial BEAS-2B cells were selected because they have been widely used to evaluate pulmonary toxicity after nanoparticle exposure [13, 21, 46]. Human U937 monocytes were selected because they have a higher passage number with stability and consistency, and they can differentiate into mature macrophages upon PMA treatment (U937*) [47]. To mimic the physiological condition in the normal human lungs, BEAS-2B cells and U937* macrophages were seeded in a ratio of either 1:1 or 9:1 in the co-culture system. These ratios were selected based on the cell composition in the normal human lung alveolar region. The 1:1 corresponds to the ratio of the total cell volume of alveolar epithelial cells (2653 µm^3^) to that of macrophages (2492 µm^3^). The 9:1 ratio corresponds to the highest ratio of the number of pneumocytes to macrophages reported in normal human lungs [48–54]. Compared to monoculture, the co-culture model provides more realistic micro-environmental conditions to mimic in vivo conditions, and it is a good in vitro method to study the role of cell–cell interactions or communications in nanoparticle-induced pulmonary toxicity [21, 55–58]. Jennifer et al. have shown that in vitro co-culture model is more sensitive as compared to the conventional monoculture in evaluating the inflammatory responses in the lower respiratory tract after nanoparticle exposure [59]. Previous studies also reported that more concordant inflammatory and fibrotic genes were expressed in the co-culture system than in monoculture when comparing gene expression in mouse lungs after nanoparticle exposure [23, 60]. In this study, our results demonstrated that in the triple co-culture model, MMP-3 and cleaved OPN released from Nano-CuO-exposed BEAS-2B and U937* cells caused the activation of MRC-5 fibroblasts, whereas direct exposure to Nano-CuO did not induce MRC-5 activation, suggesting that this triple co-culture model is sensitive and suitable for evaluating nanoparticle-induced pulmonary toxicity in vitro.

In our triple co-culture model, human lung epithelial BEAS-2B cells and U937* macrophages were seeded in the inserts of the Transwell® units, which has a diameter of 24 mm (~ 4.5 cm^2^ growth area), and were exposed to 1 µg/mL of Nano-CuO for 12 h. We chose 1 µg/mL (~ 0.22 μg/cm^2^) of Nano-CuO for this study mainly based on our cytotoxicity assay; this dose would not cause significant cytotoxicity in BEAS-2B and U937* cells in our model. The surface area of a human lung is about 100 m^2^ [61], and a previous study recommended a factor of 10 for particle uneven deposition [43]. Thus, an adult would need to have at least 0.022 g particles deposited in the lungs to reach the dose we used in vitro. Assuming an adult with a 20 L/min ventilation rate works 8 h per day for a year, the worker would have inhaled 3504 m^3^ air during work (20 L/min × 60 min/h × 8 h/day × 365 days = 3504 m^3^). Therefore, to have 0.022 g particles deposited in the lungs, the worker needs to stay in an environment with a particle air concentration of 0.063 mg/m^3^ for a year, assuming no clearance and 10% deposition rate [62] (0.022 g × 10 ÷ 3504 m^3^ = 0.063 mg/m^3^). NIOSH recommends that the exposure limit of copper fume is 0.1 mg/m^3^ (TWA) [63]. In an 0.1 mg/m^3^ particle environment and using the above assumptions for calculation, it would only take about 8 months for a worker to reach the dose we used. In addition, the concentration of particles in the workplace depends on the operation procedure, ventilation, etc., and accidental higher exposure in the workplace also cannot be ignored. Therefore, the 1 μg/mL of Nano-CuO we chose for this study is reasonable and relevant.

During pulmonary inflammation, macrophages constantly communicate with epithelial cells, the underlying mesenchymal cells, such as fibroblasts, and extracellular matrix, which form a structure called epithelial–mesenchymal cell trophic unit (EMTU), to regulate lung homeostasis and immunity against inhaled particles and other pathogens [64]. These cell–cell communications are hypothesized to be crucial in leading to pulmonary injury and fibrotic responses after exposure to respirable inhaled nanomaterials [17, 19–21, 23–25]. The roles of these cell–cell communications in fibrotic responses have been modelled in co-culture system or by using conditioned media, such as co-culture of epithelial cells and macrophages, or using conditioned media from either epithelial cells or macrophages to culture fibroblasts in Transwell® or other 3D culture systems [20, 21, 44]. For example, Salik and colleagues used conditioned media to explore the effects of MWCNTs-exposed normal human bronchial epithelial (HBE) cells on MRC-5 cells, and their results showed that conditioned media from MWCNTs-treated HBE cells induced remarkable expression of pro-fibrotic markers in MRC-5 cells, such as tenascin-c and osteopontin [20]. Pranita’s study also demonstrated that MWCNTs exposure caused inflammatory and fibrotic responses in a lung microtissues consisting of human macrophages, lung epithelial cells, and fibroblasts, reflected by increased expression of the genes, such as platelet-derived growth factor (PDGF) and COL3A1 [21]. In the present study, we employed at first a conditioned media approach, then a triple co-culture system to explore the effects of cell–cell communications of human epithelial cells and macrophages on fibroblast activation. We found that human MRC-5 fibroblasts got activated when cultured in conditioned media from Nano-CuO-exposed human lung epithelial BEAS-2B cells, U937* macrophages, or co-culture of BEAS-2B and U937* cells. Activation of unexposed MRC-5 fibroblasts was also observed in the triple co-culture system after BEAS-2B and U937* cells were exposed to Nano-CuO. Our results suggest that the cell–cell communications play important roles in Nano-CuO-induced fibrotic responses.

Next, we determined the roles of two matrix factors, MMP-3 and OPN, in Nano-CuO-induced fibrotic response. Our previous study has shown that exposure of human bronchial epithelial BEAS-2B cells to Nano-CuO caused increased expression and activity of MMP-3 [13]. OPN is a multifunctional protein that acts as a cytokine as well as an extracellular matrix, playing key roles in the formation of lung injury and fibrosis [27, 30, 65]. OPN is secreted by many kinds of cells, such as macrophages, epithelial cells, endothelial cells, and fibroblasts [30, 33, 65, 66]. It is reported that OPN is highly expressed in nanomaterial-induced fibrosis in animal models, as well as in human fibrotic diseases such as IPF [30–32, 34, 67–69]. Knockout (KO) or blocking of OPN showed protect effects against nanomaterial- or bleomycin-induced fibroblast activation and fibrosis in both in vivo and in vitro studies [16, 33, 34]. For example, Dong’s study revealed that OPN was highly induced after MWCNTs exposure in mouse lungs, and OPN knockout reduced the formation of fibrotic focus and accumulation of myofibroblasts in mouse lungs. In the cellular level, OPN stimulated differentiation of fibroblasts and production of collagen and fibronectin, which was blocked by OPN neutralizing antibodies [16]. Decreased formation of granuloma and less deposition of collagen were also observed in lungs of OPN-KO mice comparing to those in wild-type mice after MWCNTs administration [34]. In the current study, Nano-CuO exposure caused significant production of MMP-3 and OPN in BEAS-2B cells and U937* macrophages and culture of fibroblasts in either the conditioned media collected from Nano-CuO-exposed BEAS-2B and/or U937* cultures or the triple co-culture system induced their activation. Inhibition of MMP-3 expression by MMP-3 siRNA or pretreatment of MRC-5 fibroblasts with GRGDSP peptide significantly inhibited the activation and migration of MRC-5 cells, suggesting MMP-3 and OPN, as paracrine signalers, play key roles in activating MRC-5 fibroblasts.

The crosstalk of MMP-3 and OPN was also investigated in this study. OPN displays multiple biological activities through its binding motifs, such as the RGD motif or SVVYGLR integrin-binding site, to interact with a variety of cellular receptor molecules. Previous studies reported that proteolytic cleavage of OPN may either enhance or reduce the integrin-binding ability of OPN, suggesting that different cleavage sites may affect the biological activities of OPN-derived fragments or peptides [38, 70–74]. Matrix metalloproteinases (MMPs) are a large family of proteases that can degrade all kinds of extracellular matrix proteins and cleave biomolecules, such as OPN [75]. MMPs have been demonstrated to be able to cleave OPN and unmask its receptor-binding sites, which modulates the biological function of OPN by altering its integrin-binding ability [38, 71–74]. For example, a study showed that MMP-9 can cleave murine OPN at three different sites, and the OPN-p151 peptide, which is produced by the cleavage of the Gly-Leu bond (Gly^151^-Leu^152^) on the OPN, showed increased wound healing effects on fibroblasts [73]. MMP-3, another member of the MMPs family, was revealed to cleave human OPN at the Gly-Leu bond (Gly^166^-Leu^167^), which is 5 amino acids downstream of the RGD binding motif that enhances the binding ability of OPN to its receptors. And this MMP-3-cleaved OPN (40 kDa) showed a significantly enhanced ability of adhesion and migration stimulus in vitro compared with full-length OPN [38]. In this study, exposure of BEAS-2B and U937* cells to Nano-CuO caused remarkably increased expression of MMP-3 and production of MMP-3-cleaved OPN (40 kDa), and MMP-3 siRNA transfection significantly blocked the generation of MMP-3-cleaved OPN fragment as well as inhibited the activation of MRC-5 cells in the triple co-culture system, suggesting that MMP-3-cleaved OPN plays an important role in Nano-CuO-induced fibrotic responses.

Activation and differentiation of fibroblasts into myofibroblasts is a critical step in the development of pulmonary fibrosis. A variety of growth factors and cytokines can activate and stimulate fibroblast differentiation [17, 19, 69]. For example, Li et al. [76] reported that silica nanoparticles induced secretion of pro-fibrotic cytokines, such as TGF-β1, from macrophages, which further promoted the proliferation and differentiation of human lung fibroblasts MRC-5, reflected by increased expression of α-SMA and collagen I. Our previous study revealed that exposure to Nano-Ni caused activation of the TGF-β1/Smad signaling pathway and further led to pulmonary fibrosis in mouse lungs [77]. In addition to TGF-β1, OPN was shown to induce proliferation, migration, and activation of fibroblasts in mouse lungs [16, 33, 34]. In OPN-KO mice, a marked decrease in collagen deposition and reduced inflammation in the lungs were observed after SWCNT exposure compared to those in wild-type mice [34]. In another study, treatment of mice with RMV-7, an αv antibody interrupts the binding of OPN to integrins, significantly suppressed the formation of bleomycin-induced lung fibrosis, and in vitro, RMV-7 significantly repressed recombinant OPN-enhanced migration, adhesion, and proliferation of NIH3T3 murine fibroblasts [33]. In this study, Nano-CuO exposure caused increased production of MMP-3-cleaved OPN and further resulted in the activation of fibroblasts, whereas no significant upregulation of TGF-β was observed (Additional file 5). Additionally, PDGF and several other pro-inflammatory cytokines may also contribute to the progression of lung fibrosis [17, 69, 78]. For example, Salik’s study showed that conditioned media from MWCNTs-treated HBE cells caused NLRP3 inflammasome-dependent but not TGF-β-dependent fibrotic responses in fibroblasts [20].

Although OPN-induced activation of fibroblasts was confirmed in the current study and other studies [16, 33, 34], how OPN induced fibroblast activation and fibrosis still needs to be further explored. Indeed, OPN is a multifunctional factor and has been shown to interact with other factors in fibrotic responses. Previous studies demonstrated that recombinant mouse OPN enhanced platelet-derived growth factor (PDGF)-mediated cell proliferation and DNA synthesis in murine fibroblasts [33]. In addition, OPN may promote the occurrence of epithelial–mesenchymal transition, which is a cellular process playing important roles in organ development, as well as disease formation, such as fibrosis [79]. OPN-siRNA treatment can restore bleomycin (BLM)-induced decrease of E-cadherin (epithelial cell marker) and reduce the BLM-induced upregulation of vimentin (mesenchymal cell marker). OPN knockdown inhibited the upregulation of Col1A1, fibronectin, and vimentin mRNA in TGF-β1-treated A549 cells [79]. However, further study is still needed to explore the mechanism of OPN-mediated activation of fibroblasts.

Currently, arguments remain about whether the Nano-CuO-induced toxic effects are due to the nanoparticles themselves, Cu ions released from the nanoparticles, or both. Some studies demonstrated that it was particulates themselves rather than Cu ions that caused toxicity. For example, Henson et al. found that only approximately 5% of the copper became soluble after 24 h incubation of Nano-CuO in DMEM with 10% FBS at 37 °C [80], thus they concluded that Nano-CuO, but not released Cu ions, has inherent cytotoxicity in rat or human intestine cells. Another study also showed that Nano-CuO-induced oxidative stress and DNA damage in lung epithelial A549 cells was likely not explained by Cu ions released to the cell medium [10]. On the contrary, some studies showed that it was Cu ions that caused Nano-CuO-induced various effects since Cu ion chelator tetrathiomolybdate (TTM) could alleviate Nano-CuO-induced adverse effects such as oxidative stress, p38 MAPK activation, DNA damage, and cell death in HUVECs [81]. Strauch et al. also suggested that the strong intracellular Cu ions released from Nano-CuO were responsible for the high cytotoxicity and marked impact on gene expression by Nano-CuO in human bronchial epithelial cells BEAS-2B [82]. In addition, Semisch et al. reported that when 50 μg/mL of Nano-CuO in H_2_O or 1 × PBS at neutral pH were agitated (100 rpm) for 1, 4, or 7 days at 37 °C, the solubility of Nano-CuO was below 2.4%. However, in the cell culture medium DMEM with or without 10% FBS, the solubility of Nano-CuO was high, reaching 44% or 66% when 50 μg/mL of Nano-CuO were agitated for 24 h at 37 °C [83]. Therefore, Semisch et al. concluded that both copper ions released from the nanoparticles as well as the nanoparticle itself contributed to the observed toxicity in A549 and HeLa S3 cells. In this study, we found that Nano-CuO exposure caused increased expression and activity of MMP-3 and increased MMP-3-cleaved OPN in the conditioned media of lung epithelial BEAS-2B cells and U937* macrophages, which led to the activation of lung fibroblasts. And in our model, the Cu ion concentration determined by ICP-MS was only 0.15 ± 0.03 µg/mL after 1 μg/mL of Nano-CuO was incubated in complete medium (with 10% FBS) for 12 h at 37 °C. Our previous study also showed that Nano-CuO exposure caused cells to undergo epithelial–mesenchymal transition (EMT) [13]. Other groups reported that carbon nanotube (CNT) exposure induced increased expression of OPN and the development of lung fibrosis, and knocking out of OPN had a protective effect against CNT-induced pulmonary fibrosis [16, 34]. Since CNTs are not likely to ionize, but cause similar effects, as Nano-CuO, we predict that it is Nano-CuO themselves that play a major role in Nano-CuO-induced profibrotic responses and lung fibrosis although the potential role of Cu ions released from the nanoparticles was not explored in the present study. However, further studies are needed to confirm it.

## Conclusions

**Fig. 14:**
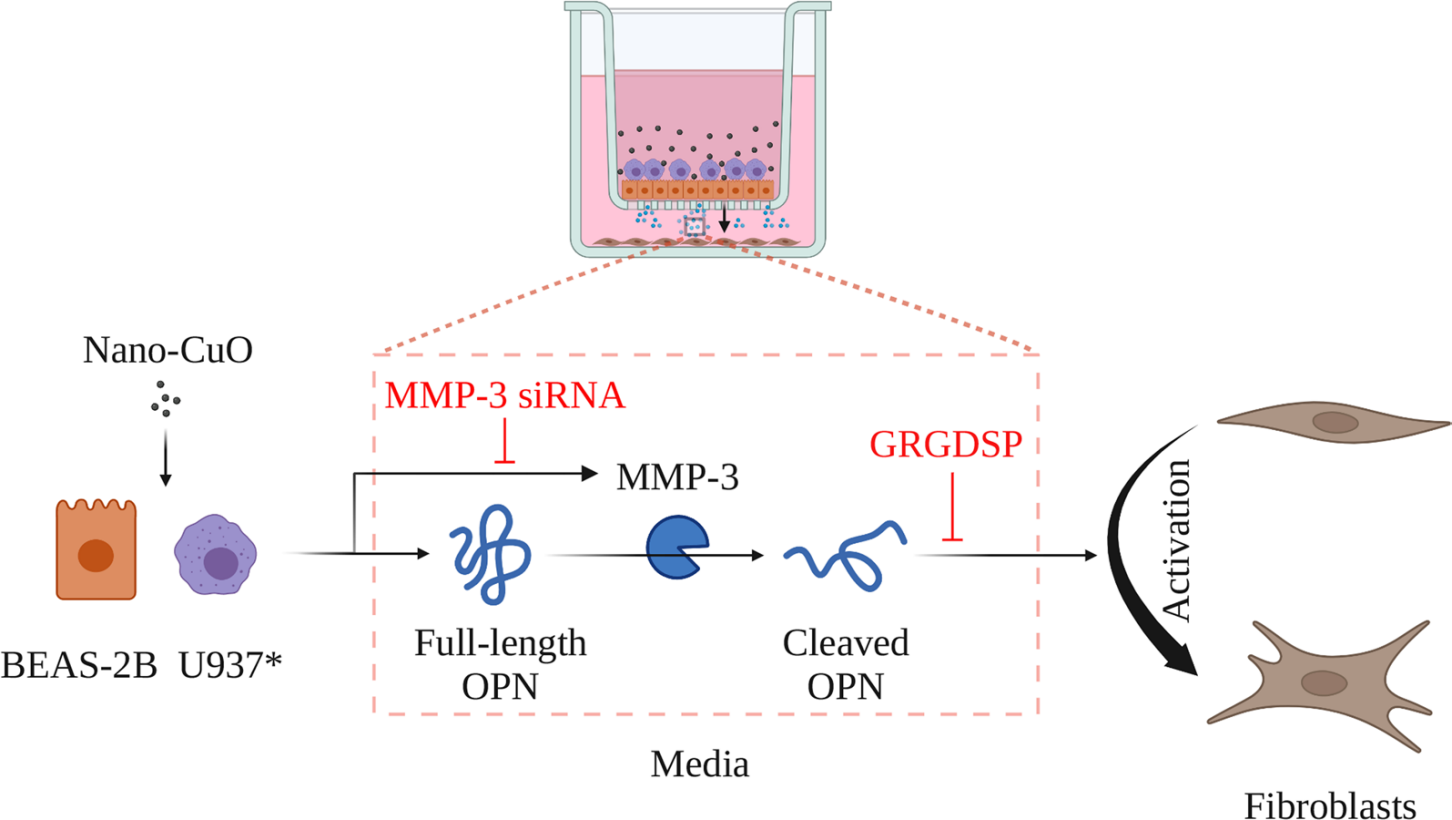
Schematic diagram of potential mechanisms of Nano-CuO-induced activation of fibroblasts. Exposure of lung epithelial BEAS-2B cells and U937* macrophages to Nano-CuO caused increased expression and activity of MMP-3 and increased production of MMP-3-cleaved OPN, leading to the activation of fibroblasts. Graphics were created with BioRender (https://BioRender.com)

Taken together, our study demonstrated that exposure of lung epithelial cells and macrophages to Nano-CuO caused increased expression and activity of MMP-3 and increased production of MMP-3-cleaved OPN, which then led to activation of fibroblasts (Fig. 14). Our results provided further understanding of pulmonary fibrosis caused by metal nanoparticle exposure (Additional file 6).

## Supplementary Information

**Additional file 1**. The effects of Nano-CuO on MMP-3 and cleaved OPN proteins in MRC-5 fibroblasts. MRC-5 cells were treated with 0.5 and 1 µg/mL of Nano-CuO for 12 h. Cells without treatment were used as control. Cell culture media were collected to detect the levels of MMP-3 (**a**) and cleaved OPN (**b**) proteins by Western blot. Equal protein loading was verified by Coomassie Brilliant Blue staining.

**Additional file 2**. Uncropped version of Western blots shown in Figure3C.

**Additional file 3**. The efficiency of MMP-3 siRNA transfection in U937* macrophages. U937* macrophages were transfected with 30 nM of MMP-3 siRNA or Negative Control No. 2 siRNA as described in the Methods. After transfection, the cells were exposed to 1 µg/mL of Nano-CuO for 12 h. Conditioned media were collected to detect the expression of MMP-3 by Western blot. Equal protein loading was verified by Coomassie Brilliant Blue staining.

**Additional file 4**. Wound healing assay for MRC-5 fibroblasts. MRC-5 fibroblasts were cultured for 48 h after a wound was created. Scale bar represents 200 µm.

**Additional file 5**. Exposure to Nano-CuO did not cause upregulation of TGF-β1 in BEAS-2B cells and U937* macrophages. BEAS-2B cells, U937* macrophages, or co-culture of BEAS-2B and U937* macrophages at the ratio of 1:1 or 9:1 were exposed to 0.5 and 1 µg/mL of Nano-CuO for 12 h. The cells without Nano-CuO exposure were used as control. After exposure, the cells were collected for protein isolation and Western blot.

**Additional file 6**. Uncropped version of Western blots shown in figures.

## Data Availability

All data and materials are included in the manuscript.

## Abbreviations

Col1A1: Collagen type 1 alpha 1
CM: Conditioned media
EMEM: Eagle’s minimum essential medium
EMT: Epithelial–mesenchymal transition
EMTU: Epithelial–mesenchymal cell trophic unit
IPF: Idiopathic pulmonary fibrosis
KO: Knockout
MMP-3: Matrix metalloproteinase-3
Nano-CuO: Copper oxide nanoparticle
OPN: Osteopontin
PMA: Phorbol 12-myristate 13-acetate
PDGF: Platelet-derived growth factor
RGD: Arg–Gly–Asp
α-SMA: α-Smooth muscle actin
SVVYGLR: Ser-Val-Val-Tyr-Gly-Leu-Arg
