# Supplementary figures and images for: MMP-3-mediated cleavage of OPN is involved in copper oxide nanoparticle-induced activation of fibroblasts
Source: Part Fibre Toxicol. 2023 May 22;20:22. doi: 10.1186/s12989-023-00532-y (PMC10201731; doi:10.1186/s12989-023-00532-y)

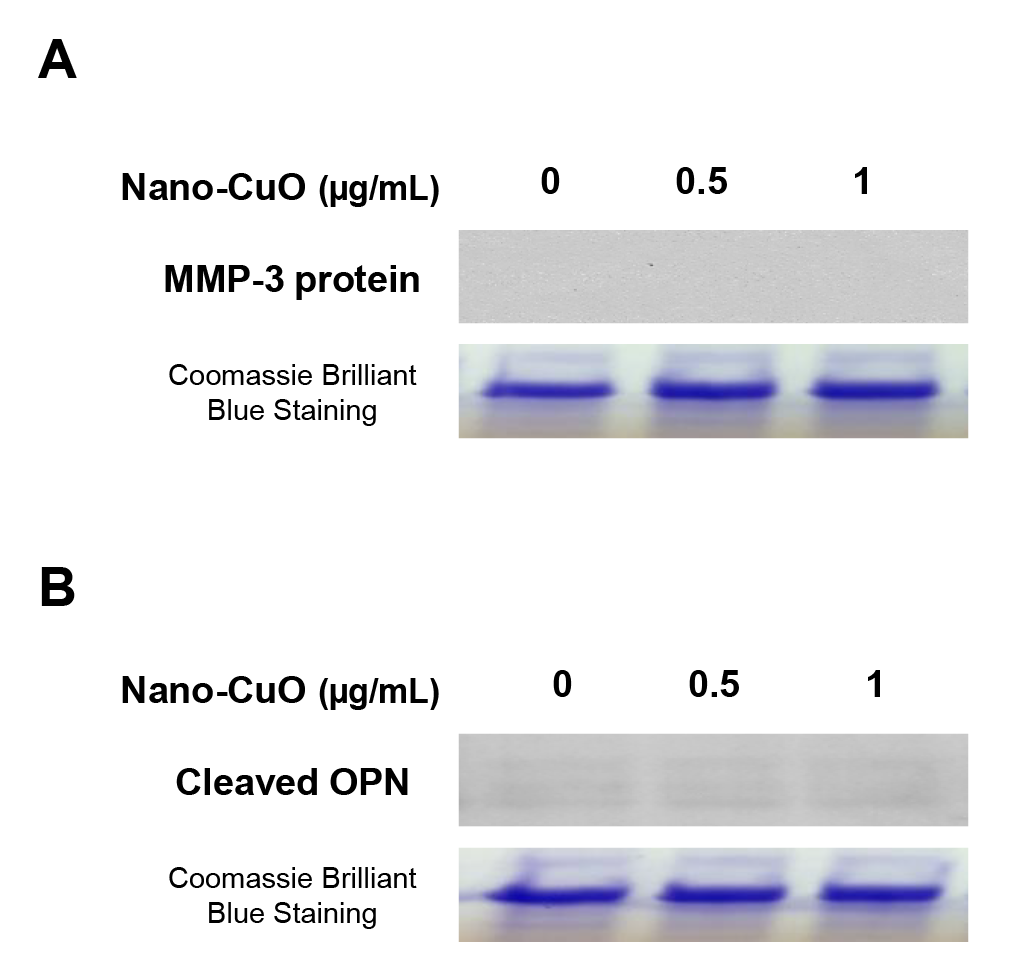

Supplement: Supplementary file 1 — Additional file 1. The effects of Nano-CuO on MMP-3 and cleaved OPN proteins in MRC-5 fibroblasts. MRC-5 cells were treated with 0.5 and 1 µg/mL of Nano-CuO for 12 h. Cells without treatment were used as control. Cell culture media were collected to detect the levels of MMP-3 (a) and cleaved OPN (b) proteins by Western blot. Equal protein loading was verified by Coomassie Brilliant Blue staining. [file 12989_2023_532_MOESM1_ESM.tif]

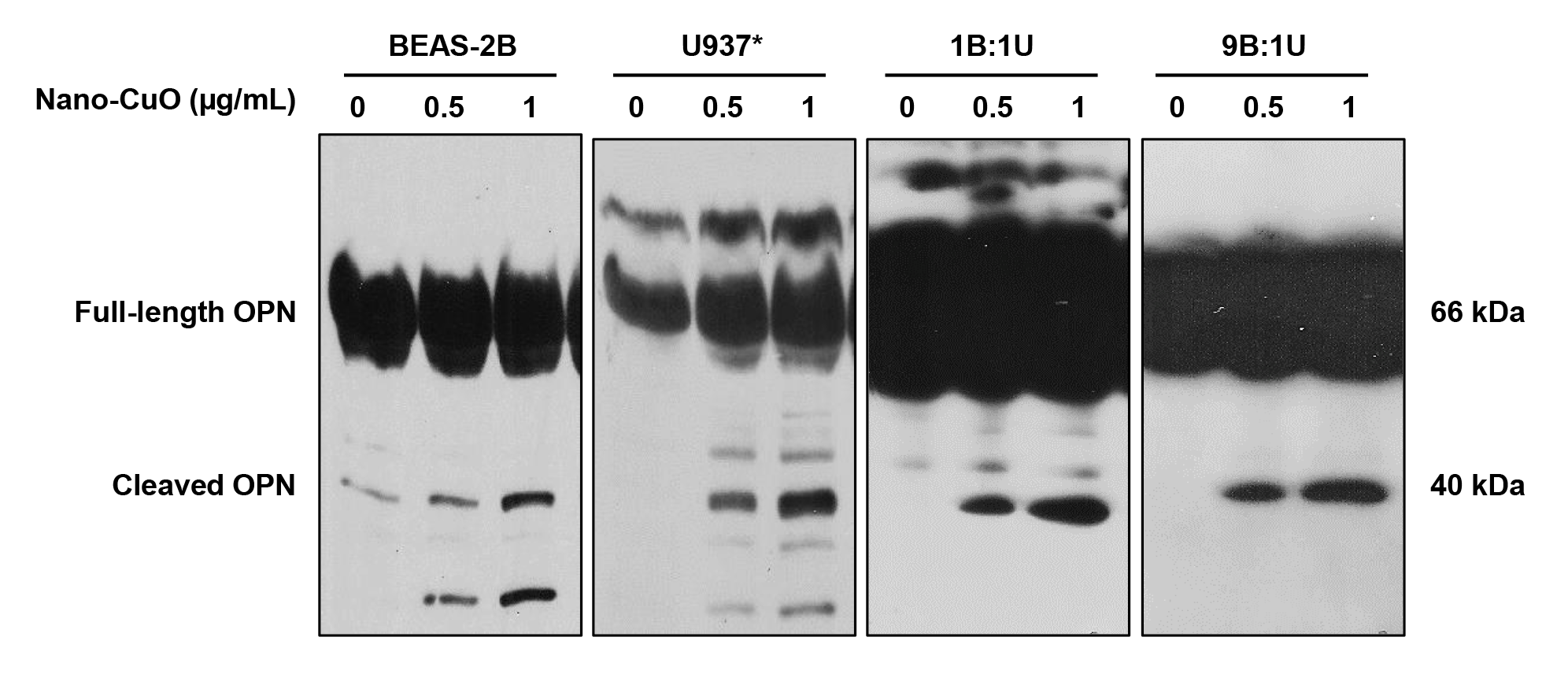

Supplement: Supplementary file 2 — Additional file 2. Uncropped version of Western blots shown in Figure3C. [file 12989_2023_532_MOESM2_ESM.tif]

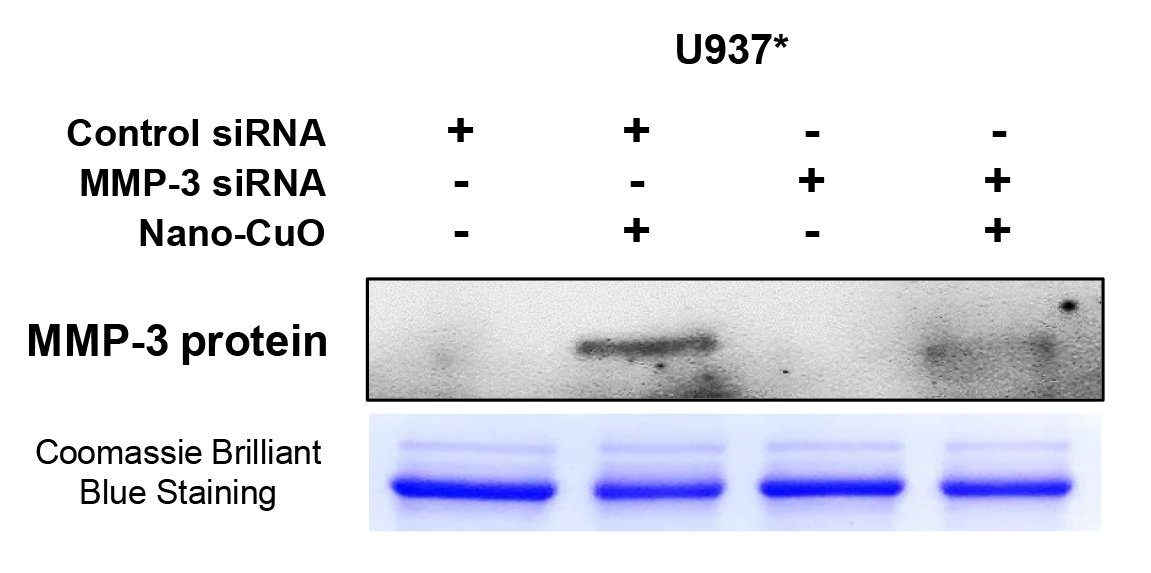

Supplement: Supplementary file 3 — Additional file 3. The efficiency of MMP-3 siRNA transfection in U937* macrophages. U937* macrophages were transfected with 30 nM of MMP-3 siRNA or Negative Control No. 2 siRNA as described in the Methods. After transfection, the cells were exposed to 1 µg/mL of Nano-CuO for 12 h. Conditioned media were collected to detect the expression of MMP-3 by Western blot. Equal protein loading was verified by Coomassie Brilliant Blue staining. [file 12989_2023_532_MOESM3_ESM.tif]

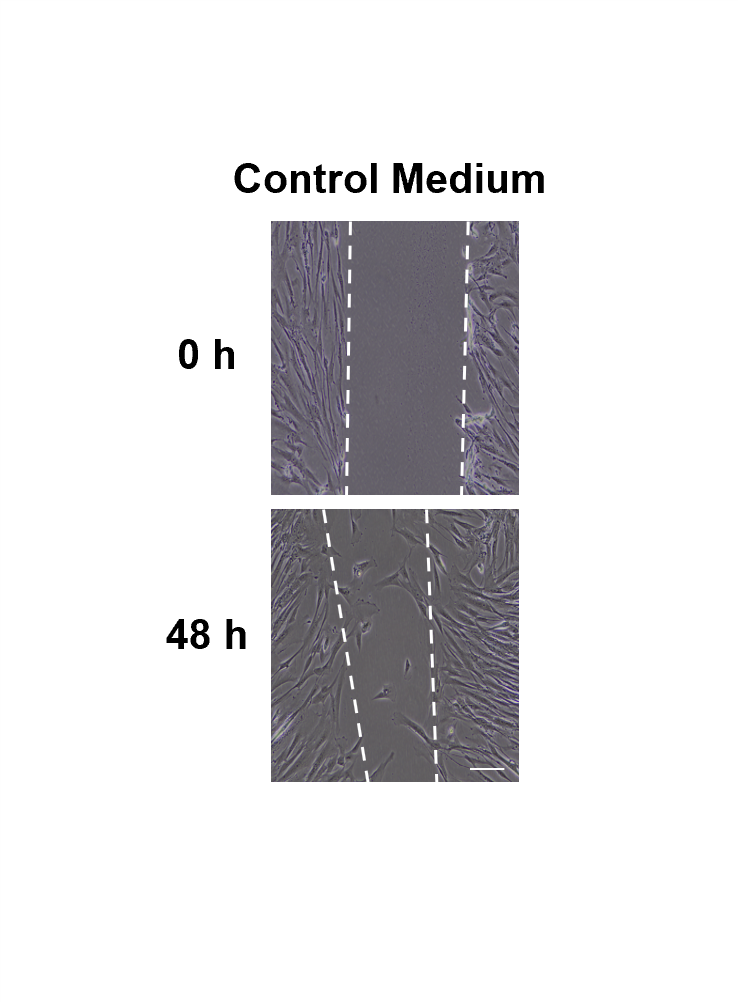

Supplement: Supplementary file 4 — Additional file 4. Wound healing assay for MRC-5 fibroblasts. MRC-5 fibroblasts were cultured for 48 h after a wound was created. Scale bar represents 200 µm. [file 12989_2023_532_MOESM4_ESM.tif]

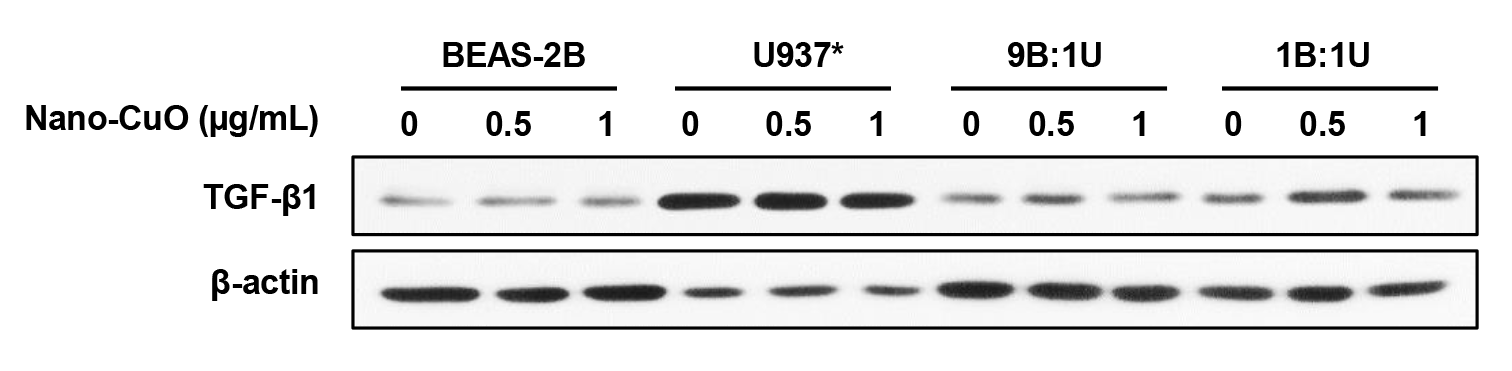

Supplement: Supplementary file 5 — Additional file 5. Exposure to Nano-CuO did not cause upregulation of TGF-β1 in BEAS-2B cells and U937* macrophages. BEAS-2B cells, U937* macrophages, or co-culture of BEAS-2B and U937* macrophages at the ratio of 1:1 or 9:1 were exposed to 0.5 and 1 µg/mL of Nano-CuO for 12 h. The cells without Nano-CuO exposure were used as control. After exposure, the cells were collected for protein isolation and Western blot. [file 12989_2023_532_MOESM5_ESM.tif]
