# Supplementary material for: MMP-3-mediated cleavage of OPN is involved in copper oxide nanoparticle-induced activation of fibroblasts
Source: Part Fibre Toxicol. 2023 May 22;20:22. doi: 10.1186/s12989-023-00532-y (PMC10201731; doi:10.1186/s12989-023-00532-y)

Figure 2

A BEAS-2B

MMP-3 protein | Coomassie Brilliant Blue Staining | **MMP-3 activity**

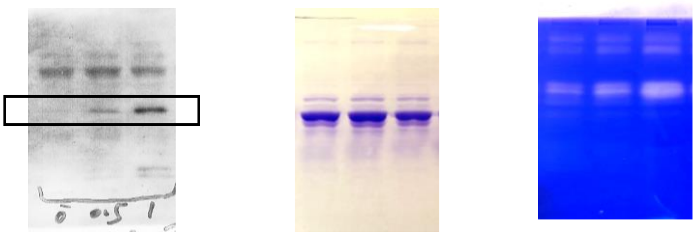

B U937*

MMP-3 protein | Coomassie Brilliant Blue Staining | **MMP-3 activity**

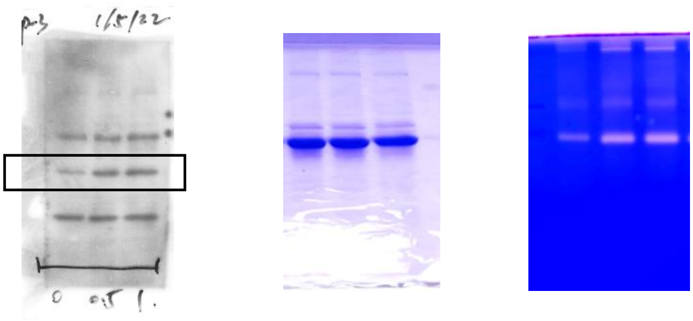

E

MMP-3 activity

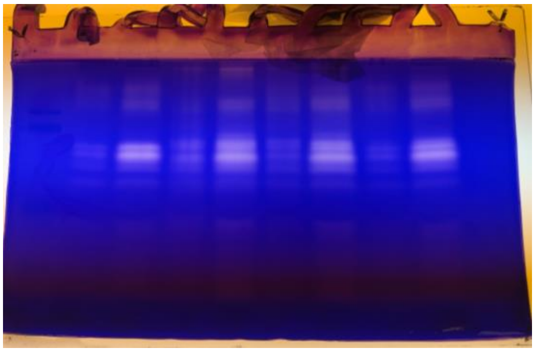

Figure 3

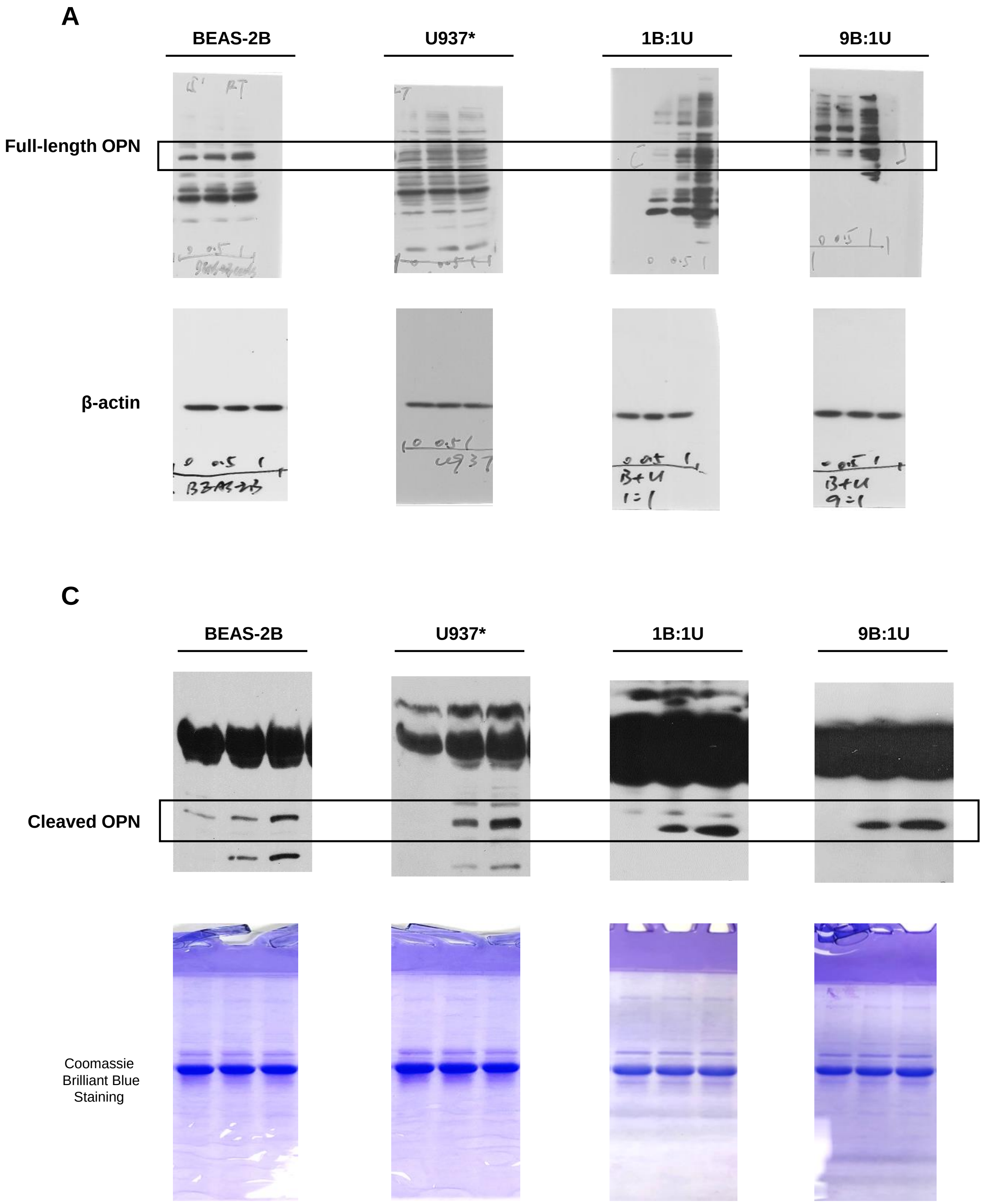
A
BEAS-2B
U937*
1B:1U
9B:1U
Full-length OPN
β-actin
C
BEAS-2B
U937*
1B:1U
9B:1U
Cleaved OPN
Coomassie Brilliant Blue Staining

Figure 4

A BEAS-2B

Cleaved OPN

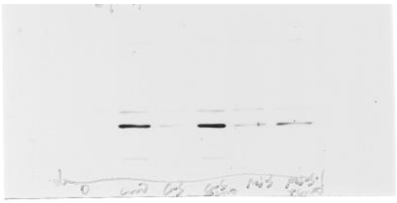

Coomassie
Brilliant Blue
Staining

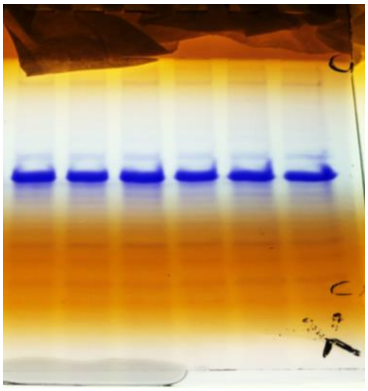

C U937*

Cleaved OPN

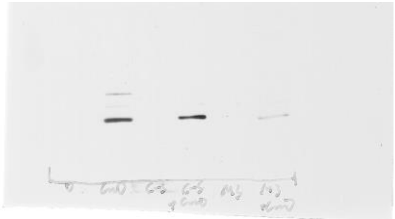

Coomassie
Brilliant Blue
Staining

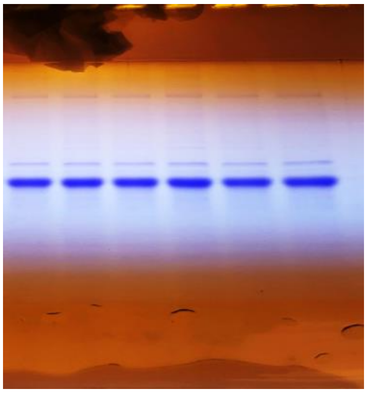

Figure 5

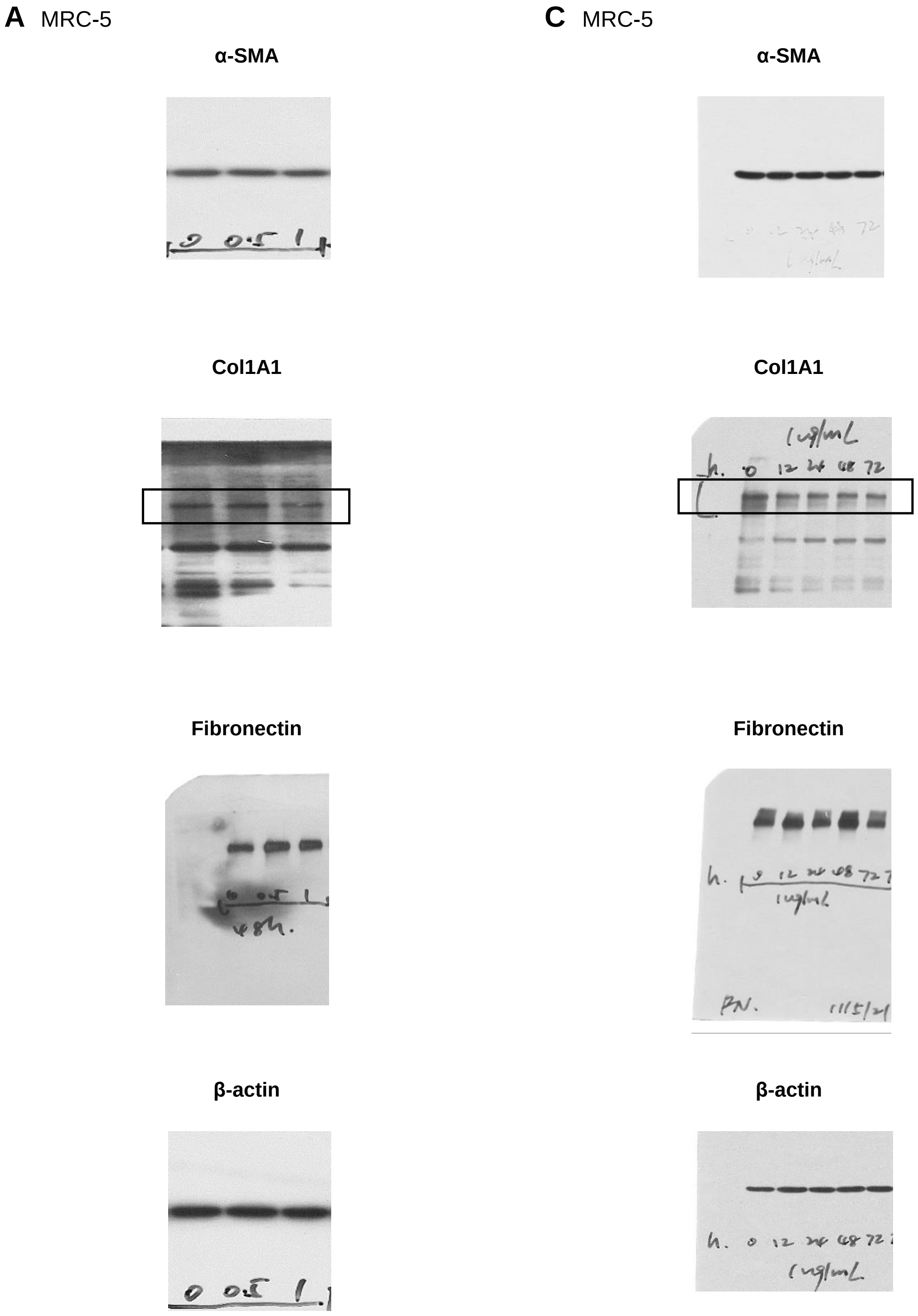
A
MRC-5
α-SMA
Col1A1
Fibronectin
β-actin
C
MRC-5
α-SMA
Col1A1
Fibronectin
β-actin

Figure 6

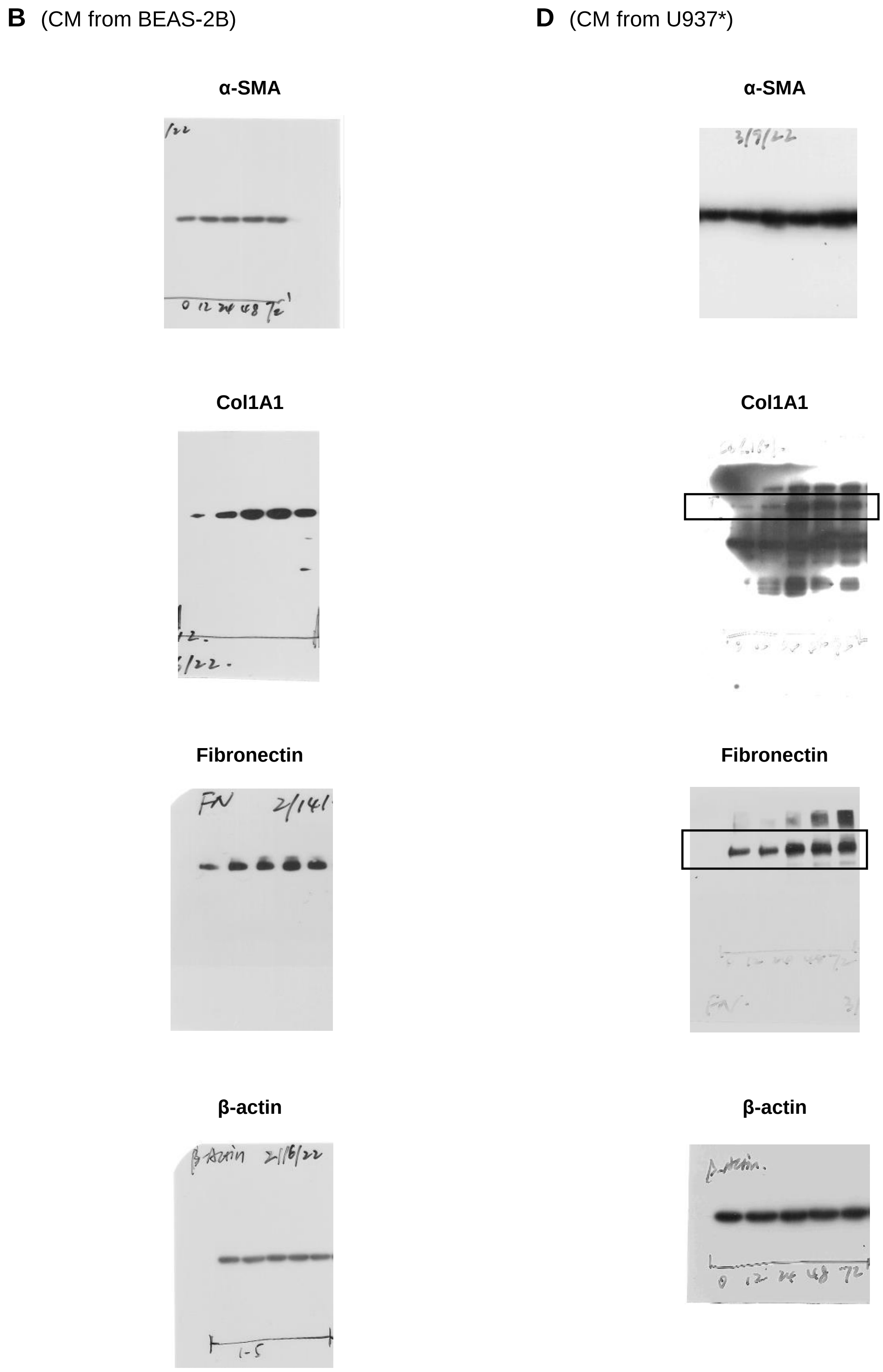
B (CM from BEAS-2B)
D (CM from U937*)
α-SMA
α-SMA
Col1A1
Col1A1
Fibronectin
Fibronectin
β-actin
β-actin

Figure 8

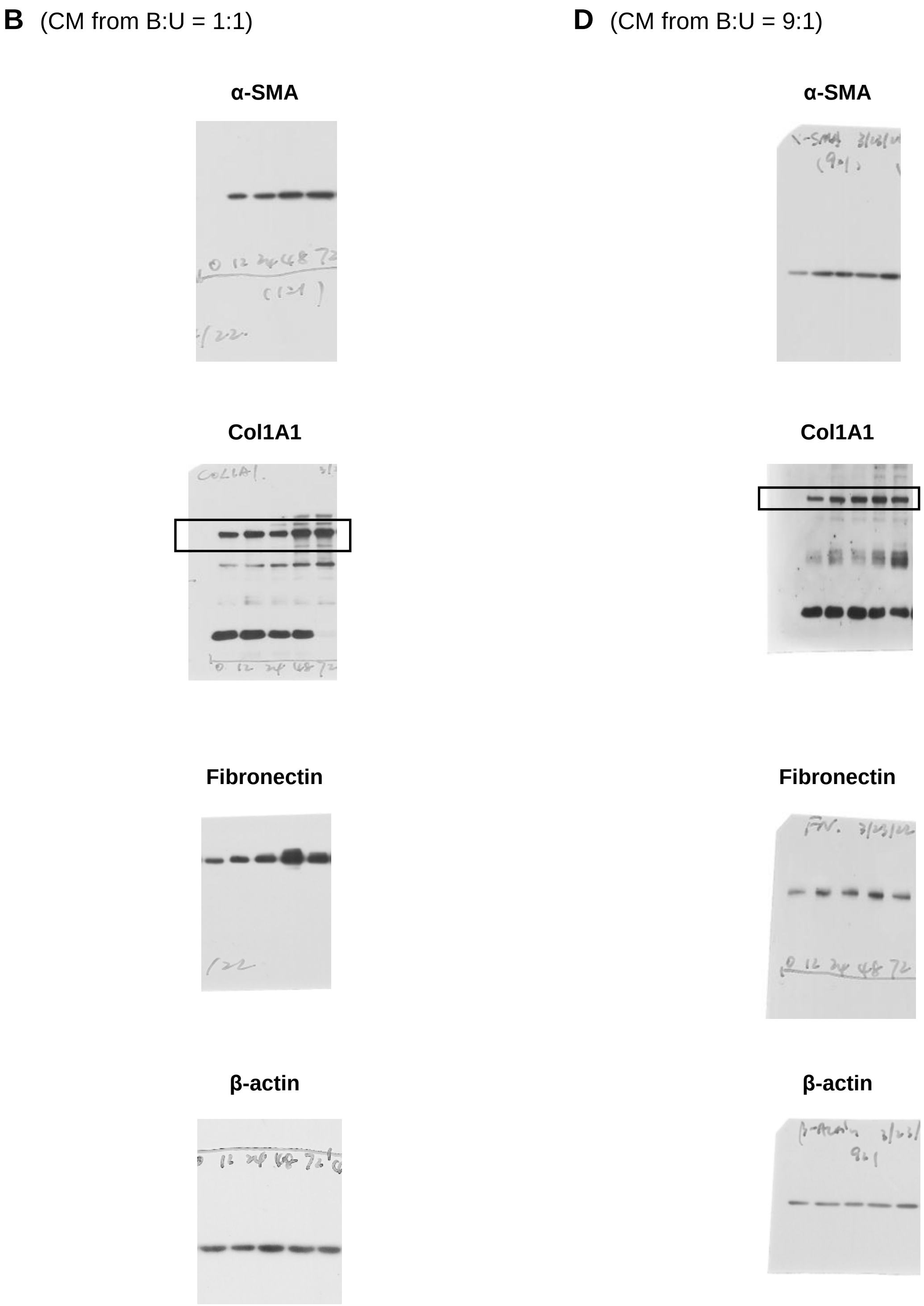
B (CM from B:U = 1:1)
D (CM from B:U = 9:1)
α-SMA
α-SMA
Col1A1
Col1A1
Fibronectin
Fibronectin
β-actin
β-actin

Figure 9

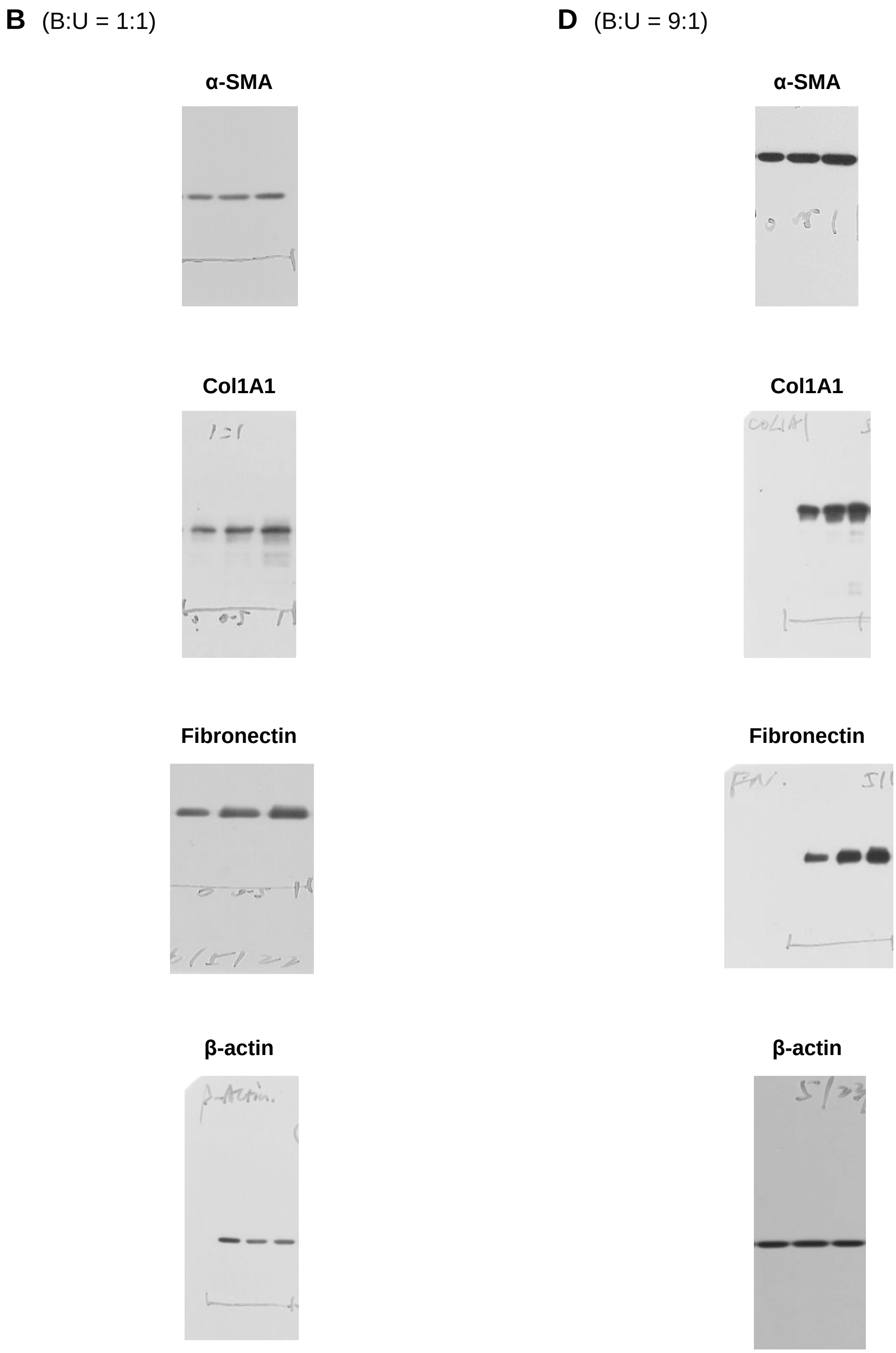
B (B:U = 1:1)
D (B:U = 9:1)
α-SMA
Col1A1
Fibronectin
β-actin
α-SMA
Col1A1
Fibronectin
β-actin

Figure 10

A (B:U = 1:1)

α-SMA

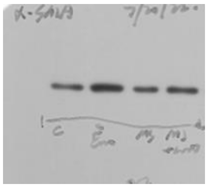

Col1A1

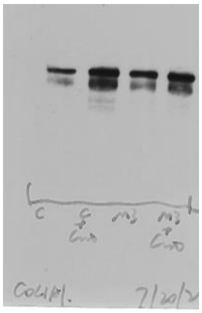

Fibronectin

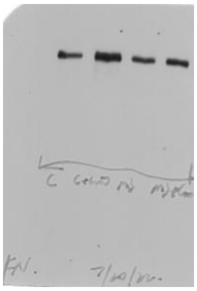

β-actin

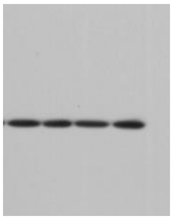

C (B:U = 9:1)

α-SMA

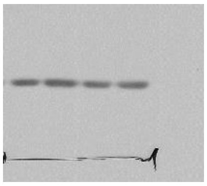

Col1A1

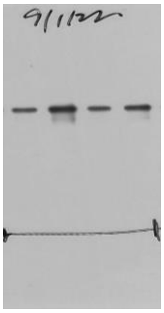

Fibronectin

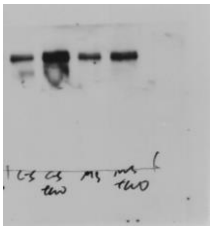

β-actin

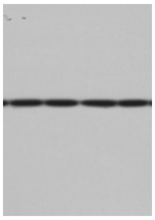

Figure 12

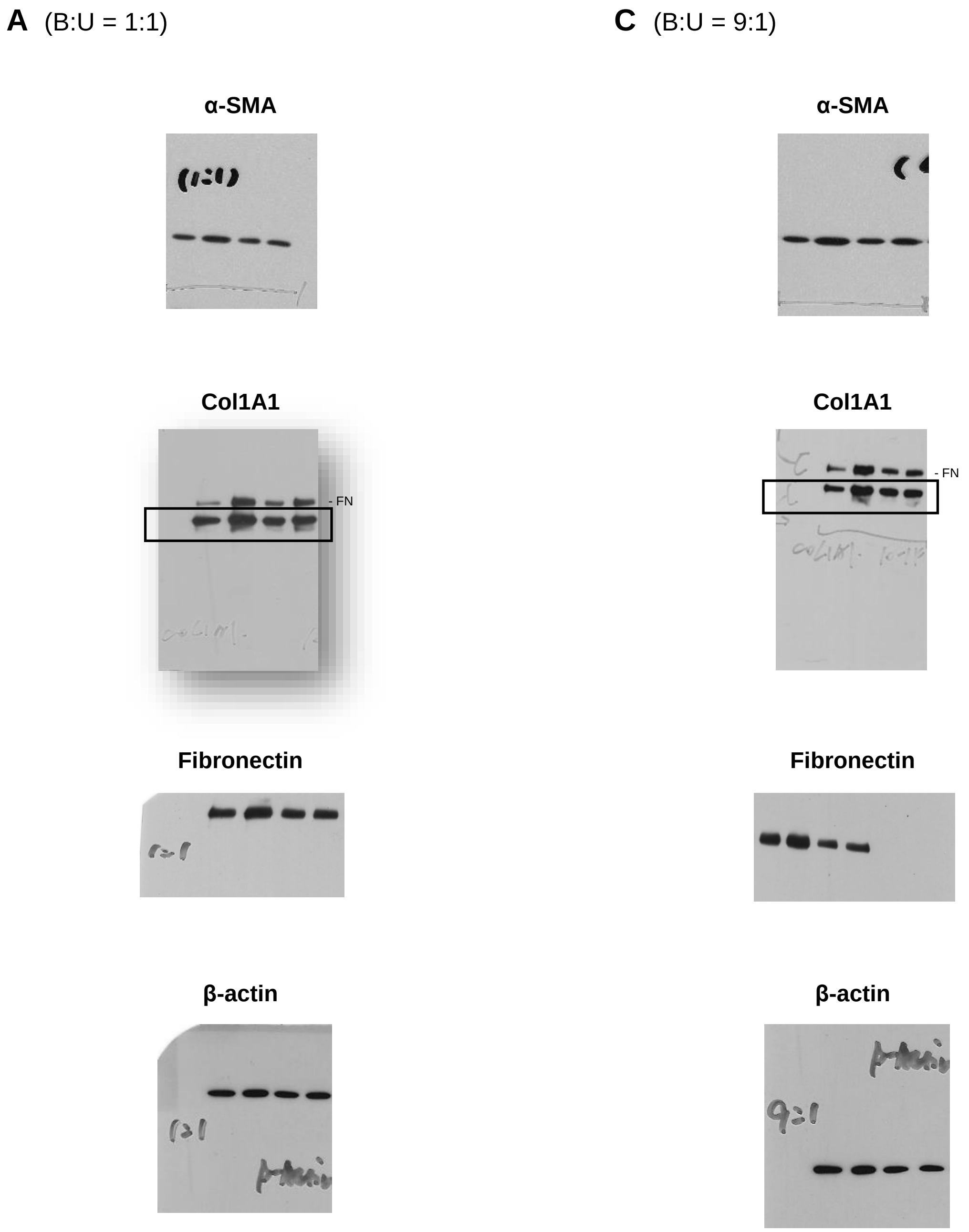

A (B:U = 1:1)
C (B:U = 9:1)
α-SMA
α-SMA
Col1A1
Col1A1
- FN
- FN
Fibronectin
Fibronectin
β-actin
β-actin

Additional file 1

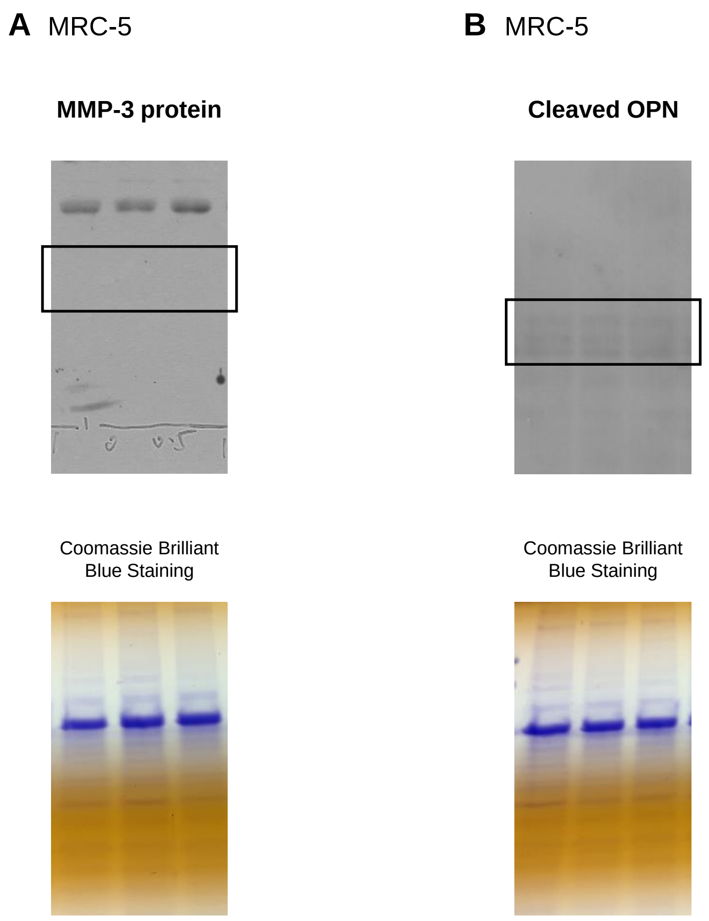

Additional file 3

MMP-3 protein

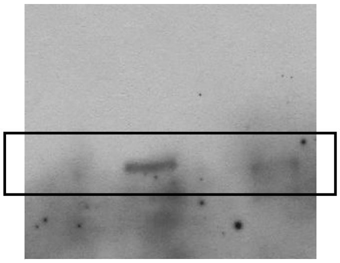

Coomassie Brilliant
Blue Staining

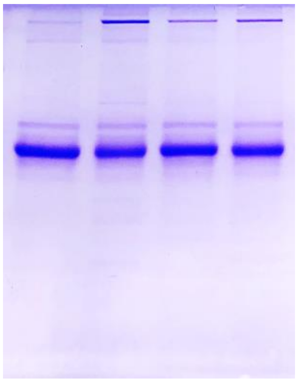

Additional file 5

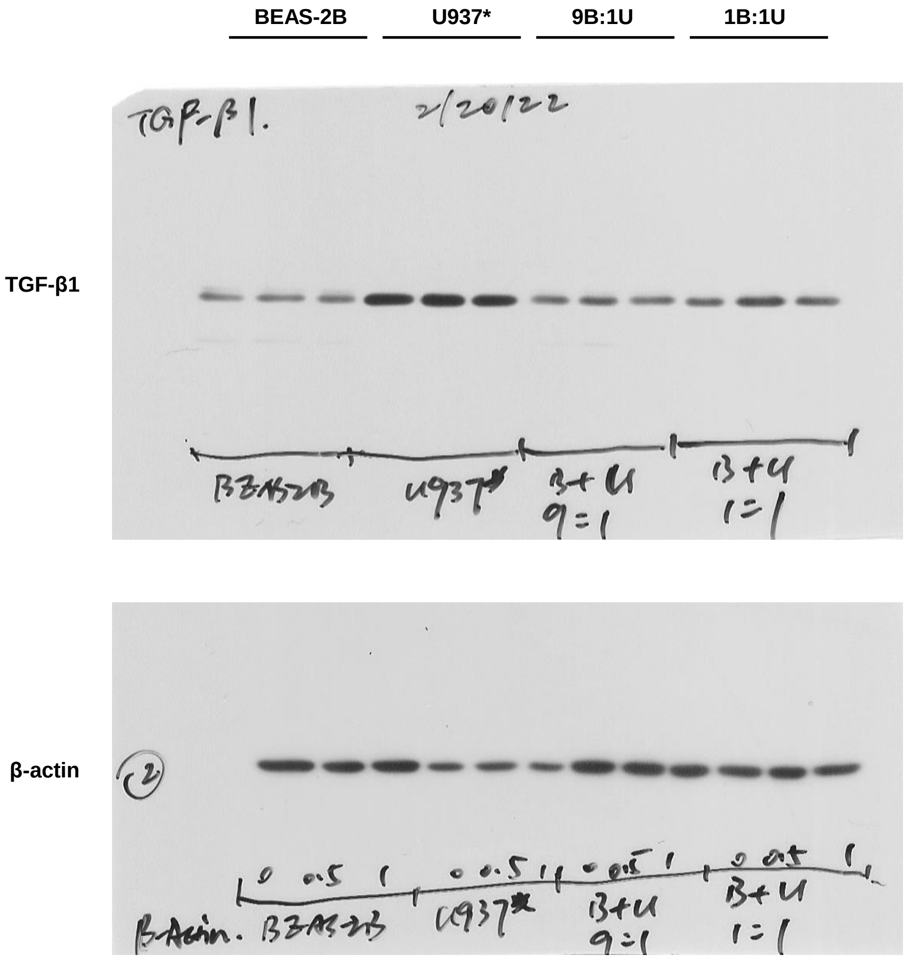

Supplement: Supplementary file 6 — Additional file 6. Uncropped version of Western blots shown in figures. [file 12989_2023_532_MOESM6_ESM.pdf]
